# Hybrid Chitosan/PCL Shape Memory Scaffolds with Potential for Bone Regeneration and Infection Resistance

**DOI:** 10.1021/acsbiomaterials.5c01160

**Published:** 2025-08-21

**Authors:** Damion T. Dixon, Ainsley G. Shields, Shane J. Stafslien, Lyndsi Vander Wal, Melissa A. Grunlan

**Affiliations:** † Department of Biomedical Engineering, 14736 Texas A&M University, College Station, Texas 77843, United States; ‡ Department of Coatings and Polymeric Materials, 3323 North Dakota State University, Fargo, North Dakota 58108, United States; § Department of Materials Science and Engineering, 14736 Texas A&M University, College Station, Texas 77843, United States; ∥ Department of Chemistry, 14736 Texas A&M University, College Station, Texas 77843, United States

**Keywords:** chitosan, antimicrobial, bone scaffold, shape memory polymer, poly(ε-caprolactone), bone regeneration, tissue engineering

## Abstract

We have previously developed a regenerative engineering approach to repair irregularly shaped craniomaxillofacial bone defects utilizing “self-fitting” shape memory polymer (SMP) scaffolds based on cross-linked poly­(ε-caprolactone) (PCL). However, a slow rate of degradation may hinder neotissue infiltration, and a lack of innate antimicrobial activity creates vulnerability to postoperative infection stemming from biofilm formation. Introduction of chitosan (CS), a hydrophilic natural polymer with known antimicrobial behavior, to PCL SMP scaffolds could provide a synergistic combination of desirable properties. Herein, for the first time, we report the development of hybrid (i.e., formed from a synthetic and a naturally derived polymer) CS/PCL SMP scaffolds. A series of eight highly porous PCL/CS-*graft*-PCL scaffolds were formed as semi-interpenetrating networks (semi-IPNs) using cross-linkable PCL-diacrylate (PCL-DA) and thermoplastic CS-*graft*-PCL copolymers. Scaffold CS content was tuned by graft copolymer composition and wt % ratio to PCL-DA. A solvent-cast particulate leaching process produced scaffolds with highly interconnected macropores (∼240 μm), which is conducive to osteogenesis. Owing to sufficient retention of PCL crystallinity, all hybrid scaffolds retained excellent shape memory and robust mechanical behavior. Compared with PCL scaffold controls, hybrid scaffolds of sufficient CS content exhibited faster rates of in vitro degradation, which is favorable to osteoinductivity. Accelerated degradation was related to increased hydrophilicity and phase separation effects. Hybrid scaffolds also displayed an ability to reduce *C. albicans* biofilm formation by both direct and indirect contact, compared with PCL scaffolds.

## Graphical abstract

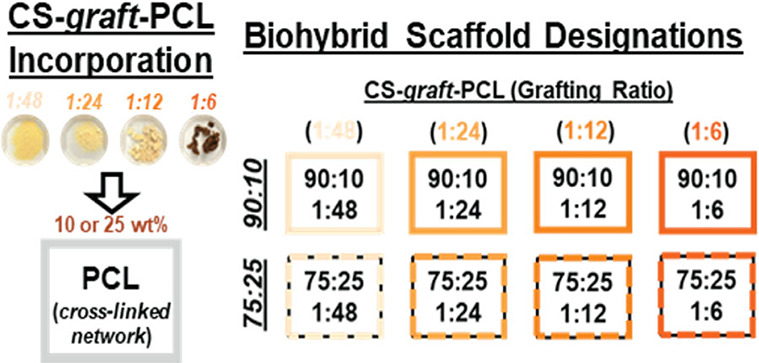

## 1 Introduction

Craniomaxillofacial (CMF) bone defects stem from a wide range of clinical scenarios (e.g., trauma, congenital deformities, and craniotomy), often resulting in defects with complex, irregular geometries.^1^ Autografting is the clinical “gold standard” for repair, though difficulties in fitting these rigid autologous tissues tightly within irregular defects can lead to inadequate tissue contact (i.e., insufficient contact between the graft and borders of the defect), ultimately causing graft resorption.^2,3^ Moreover, in situ curing bone substitutes (e.g., putties and cements) exhibit postcure shrinkage, along with other disadvantages, such as low porosity, brittleness, and nondegradability, which are not ideal for bone healing.^4,5^ CMF defect repair is further hindered by postoperative infection, prompting subsequent surgical procedures to remove the infected tissue if microbial biofilm cannot be eliminated by the body or by antibiotics.^6,7^ Therefore, a regenerative engineering approach requires a scaffold to be conformally fitting (for osseointegration) and to possess antimicrobial efficacy. These scaffolds should also be osteoinductive (i.e., support neotissue infiltration), which necessitates adequate porosity and pore interconnectivity, as well as robust degradation rates.^8^ Thus, an off-the-shelf regenerative scaffold capable of conformally fitting within an irregular defect while also providing mechanical robustness, pro-osteoconductivity, and antimicrobial character would offer considerable advantages.

We have previously reported on “self-fitting” shape memory polymer (SMP) scaffolds based on biodegradable linear-poly­(ε-caprolactone)-diacrylate (PCL-DA, *M*_n_ ∼ 10 kg mol^–1^) for treating irregular CMF bone defects.^9–11^ These highly porous (∼70%) scaffolds are prepared by photocuring macromer solutions over a fused salt template (i.e., solvent-cast particulate leaching, SCPL), resulting in scaffolds with interconnected macropores (*d* ∼ 220 μm). For these scaffolds, the PCL lamellae act as switching segments, and the covalent cross-links serve as netpoints. The “self-fitting” behavior is actuated by the melt transition temperature (*T*_m_ or “*T*_trans_”) of PCL (∼55 °C), such that when heated (*T* > *T*_trans_) (i.e., exposed to warm saline), scaffolds become soft and malleable due to the lamella melting. This allows the scaffolds to be press-fit into the irregular geometries of CMF bone defects, wherein shape recovery drives expansion toward the defect boundary, providing a conformal fit. Upon cooling to body temperature (*T* < *T*_trans_), the lamellae recrystallize, causing the scaffolds to regain their rigidity within the defect and lock in their new shape (i.e., shape fixity). These PCL scaffolds were mechanically robust, characterized by a compressive modulus in the range of trabecular bone and an absence of brittleness. Subsequently, we have shown that mechanical rigidity can be improved and the rate of biodegradation increased with a semi-interpenetrating network (semi-IPN) design based on cross-linked PCL-DA and thermoplastic (i.e., non-cross-linked) poly­(l-lactic acid) (PLLA).^12–14^ Should prolonged irrigation at the surgical site be necessary for increased “fitting time”, to improve tissue safety, the *T*_m_ can be reduced to ∼45 °C with a *star*-PCL macromer architecture (*M*_n_ ∼ 10k g mol^–1^).^15,16^ Nonetheless, these PCL-based SMP scaffolds lack the innate ability to mitigate infection, which may limit their utility in bone tissue repair. Postoperative infections, particularly those resulting in stubborn biofilm formation, are a major limiting factor in the success of CMF bone defect repair.^6,7^ Thus, the development of new strategies to limit biofilm formation on regenerative implants has become paramount.^17,18^

An emerging strategy to impart antimicrobial properties to PCL-based scaffolds is through their enrichment with natural polymers, such as chitosan (CS).^19–21^ CS is a polycationic, linear polysaccharide obtained through the deacetylation of chitin, the main constituent of crustacean shells, insect exoskeletons, and the cell wall of fungi.^22^ CS possesses broad-spectrum antibacterial and antifungal activities while maintaining minimal toxicity toward normal mammalian cells, making it well studied for wound healing applications.^23,24^ In addition to its innocuous degradation products, CS demonstrates anti-inflammatory properties while promoting cell adhesion and proliferation, which contribute to enhanced tissue regeneration and reduced scar formation.^25,26^ CS has been used in FDA-approved wound dressings and is generally recognized as safe (GRAS), making it a potentially useful regenerative engineering biomaterial, particularly for bone, garnering significant recent interest for its use in scaffold development.^27,28^ Unfortunately, processing of CS is limited owing to its poor solubility in common organic solvents and neutral aqueous media; thus, CS derivatives with enhanced solubility have become increasingly studied.^29,30^ CS has been modified through the grafting of synthetic polymers onto its backbone and studied for various applications.^31–34^ Hence, the graft copolymerization of CS with PCL is a promising way to exploit the favorable characteristics of each.^25,29^ Alone, such CS-*graft*-PCL copolymers are still difficult to process but have been physically blended with PCL to form scaffolds for bone regeneration.^35^ We hypothesized that combining CS-*graft*-PCL copolymers and PCL in a semi-IPN design could afford “hybrid” scaffolds with beneficial properties. Thus, scaffolds were formed from cross-linked PCL-DA and uncross-linked CS-*graft*-PCL (PCL/CS-*graft*-PCL) to enhance the rate of degradation and to impart antimicrobial function while maintaining shape memory behavior and robust mechanical properties provided by PCL.

Herein, CS-*graft*-PCL copolymers of varying CS content were synthesized by grafting PCL to the hydroxyl groups present on the backbone of CS in a ring-opening polymerization reaction (Figure 1). The reaction feed molar ratio (FMR) of CS (based on glucosamine units) to ε-caprolactone was systematically tuned, producing four distinct CS-*graft*-PCL copolymers (i.e., 1:6, 1:12, 1:24, and 1:48). Eight scaffold compositions were formed by combining PCL-DA and each CS-*graft*-PCL copolymer at 90:10 and 75:25 wt % via the established SCPL technique (Figure 2). These PCL/CS-*graft*-PCL semi-IPN scaffolds were directly compared to a scaffold control formed from only PCL-DA (i.e., without CS-*graft*-PCL). Scaffolds were thoroughly characterized in terms of their pore, thermal, shape memory, mechanical, and degradative properties. Analogous solid films were used to determine hydrophilicity via contact angle analysis and phase separation. Films were also utilized to assess the resistance to biofilm growth and retention of the fungus *C. albicans*.

**Figure 1:**
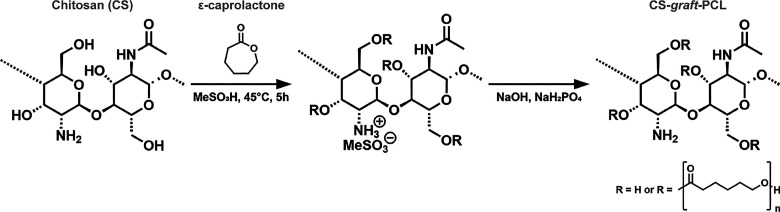
Chemical synthesis of CS-*graft*-PCL copolymers.

**Figure 2:**
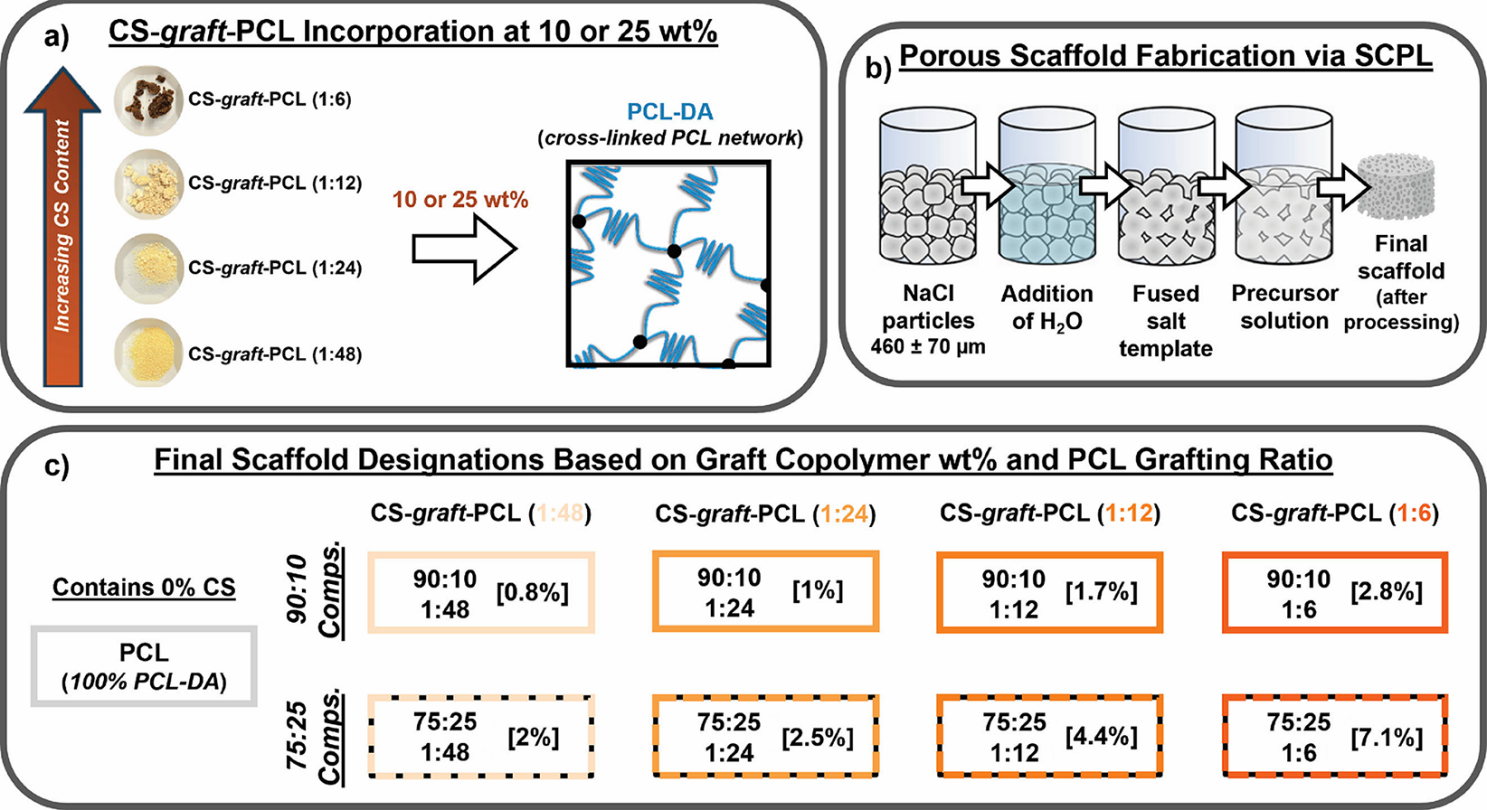
(a) Preparation of the PCL/CS-*graft*-PCL scaffolds. For CS-*graft*-PCL copolymers, ratios (1:6, 1:12, 1:24, and 1:48) represent FMRs of glucosamine units in chitosan to ε-caprolactone. Semi-IPNs were formed by combining PCL-DA and CS-*graft*-PCL at either 90:10 or 75:25 wt %. (b) Schematic of the SCPL process to fabricate porous scaffolds to achieve interconnected macropores. (c) Final scaffold designations. “[%]” represents final wt % of CS in scaffolds.

## 2 Materials and Methods

### 2.1 Materials

CS (purified powder, *M*_w_ = 15 kg mol^–1^ per manufacturer, % degree of deacetylation (DDA) = 86%, calculated from ^1^H NMR spectroscopy) was purchased from Polysciences, Inc. (Warrington, PA, USA) and vacuum-dried (50 °C, 30 in. Hg) for 24 h prior to use. Deuterium oxide (D_2_O), deuterium chloride (DCl), methanesulfonic acid (MeSO_3_H), ε-caprolactone, potassium phosphate monobasic (KH_2_PO_4_), sodium hydroxide (NaOH), poly­(ε-caprolactone) diol (PCL-diol, *M*_n_ ∼ 10 kg mol^–1^ per manufacturer), reagent-grade dichloromethane (DCM), 4-(dimethylamino)-pyridine (DMAP), triethylamine (Et_3_N), acryloyl chloride, ethyl acetate, potassium carbonate (K_2_CO_3_), anhydrous magnesium sulfate (MgSO_4_), deuterated chloroform (CDCl_3_), sodium chloride (NaCl), 2,2-dimethoxy-2-phenylacetophenone (DMP), 1-vinyl-2-pyrrolidinone (NVP), reagent alcohol (ethanol), tetrahydrofuran (THF), and polystyrene (PS) were purchased from Sigma-Aldrich (St. Louis, MO, USA). Phosphate-buffered saline (PBS, 1X solution, Ca, Mg, pH 7.4) was purchased from Corning, Inc. (Corning, NY, USA). Deionized (DI) water (18 MΩ) was obtained from an in-house purification system. All solvents were dried over 4 Å molecular sieves (Sigma-Aldrich), and all reagents were vacuum-dried (room temperature [RT], overnight [ON], 30 in. Hg) prior to use.

### 2.2 Synthesis

Reactions were conducted under positive nitrogen (N_2_) pressure in oven-dried (120 °C) glassware utilizing Teflon-covered magnetic stir bars to agitate the reaction mixtures. Polymer structures (including the *M*_n_ and % acrylation of PCL-DA) were confirmed by ^1^H NMR spectroscopy (Avance NEO 400 MHz spectrometer) operating in Fourier transform mode with CDCl_3_ as the standard.

CS-*graft*-PCL copolymers were synthesized by grafting ε-caprolactone monomers onto CS via ring-opening polymerization as described elsewhere, with minimal modification.^29^ In a typical reaction, thoroughly dried CS (1.0 g, 6.0 mmol of glucosamine units, considering % DDA) and MeSO_3_H (15 mL) were combined in a 100 mL round-bottom flask (rbf) equipped with a rubber septum, and the mixture was stirred continuously at 45 °C until the CS had completely dissolved (∼30 min). Following a 3 min N_2_ purge, ε-caprolactone (32.8 g, 287.4 mmol, 48 equiv) was injected into the flask. The reaction mixture was stirred at 45 °C under positive N_2_ pressure for ∼5 h before being precipitated into a solution containing precisely 100 mL of 0.2 M KH_2_PO_4_, 16 mL of 10 M NaOH, and 100 g of crushed ice. The precipitate was collected by vacuum filtration and washed several times with DI water, rendering the pH neutral. The resulting graft copolymer was vacuum-dried (RT, ON, 30 in. Hg), labeled as CS-*graft*-PCL (1:48), and stored in a desiccator until further use. Likewise, CS-*graft*-PCL (1:24), CS-*graft*-PCL (1:12), and CS-*graft*-PCL (1:6) were synthesized with variations in the reaction FMR (i.e., glucosamine units in CS to ε-caprolactone).

PCL-DA was prepared by converting the terminal hydroxyl groups of PCL-diol (*M*_n_ ∼ 10 kg mol^–1^) to photosensitive acrylate groups by reacting with acryloyl chloride, similar to that previously reported.^10^ Briefly, PCL-diol (20.0 g) and DCM (120 mL) were combined in a 250 mL rbf apparatus equipped with a rubber septum and stirred until the PCL-diol was completely dissolved. DMAP (6.6 mg) was added as a catalyst and allowed to fully dissolve, followed by a 3 min N_2_ purge and the sequential dropwise addition of Et_3_N (0.56 mL, 4.0 mmol) and acryloyl chloride (0.65 mL, 8.0 mmol). The reaction was stirred at RT under positive N_2_ pressure for 30 min and subsequently refluxed at 55 °C for ∼20 h. The solvent was removed using a rotary evaporator, and the crude product was dissolved in ethyl acetate (135 mL) prior to gravity filtration. The solvent was once again removed, and the isolated PCL-DA was redissolved in DCM (135 mL), washed with 13.5 mL of 2 M K_2_CO_3_, and placed in a separatory funnel ON. After separation, the organic layer was collected, dried with MgSO_4_ (∼5 g), gravity-filtered, dried using a rotary evaporator, and finally vacuum-dried (RT, ON, 30 in. Hg) to obtain a purified product (∼80% yield). ^1^H NMR agreed with previously reported results (*M*_n_ ∼ 10 kg mol^–1^ and >90% acrylation).^15,16^

### 2.3 CS-*graft*-PCL Characterization

Prior to scaffold fabrication, CS-*graft*-PCL copolymers were characterized to determine their chemical composition, molecular weights, and CS content (wt %). Detailed methods are provided in the Supporting Information, while a summary of relevant properties is outlined in Table 1.

**Table 1:** Characterization of the CS-*graft*-PCL Copolymers

sample	FMR^a^	yield^b^ (%)	*M*_n_ ^c^ (kg mol^–1^)	*M*_w_ ^c^ (kg mol^–1^)	PDI^c^	CS^d^ (%)
CS-*graft*-PCL (1:48)	1:48	91.12	10.76	26.94	1.90	8.11 ± 1.36
CS-*graft*-PCL (1:24)	1:24	88.36	14.10	31.29	2.01	9.99 ± 0.50
CS-*graft*-PCL (1:12)	1:12	85.94	16.40	32.99	2.22	17.38 ± 0.63
CS-*graft*-PCL (1:6)	1:6	54.46	18.03	34.16	2.50	28.38 ± 2.76

a Feed molar ratio of glucosamine units in chitosan (*M*_w_ = 15 kg mol^–1^) to ε-caprolactone added to each reaction.

b $\mathrm{Yield}\left(\%\right)=\frac{{\mathrm{mass}}_{\mathrm{Chitosan}-graft-\mathrm{PCL}}}{{\mathrm{mass}}_{\mathrm{chitosan}}+{\mathrm{mass}}_{\varepsilon -\mathrm{caprolactone}}}\times 100$ .

c Measured by GPC with PS used as the standard.

d Determined via TGA.

### 2.4 Scaffold Fabrication

Scaffolds with highly interconnected macropores were prepared via SCPL according to previously established procedures.^9,10^ Sieved NaCl particles (10 g, 460 ± 60 μm) were placed inside 20 mL scintillation vials (I.D. = 25 mm). DI water (7.5 wt %) was then added in four equal portions with mechanical stirring (utilizing a smooth glass rod) between each addition. After compaction, the vials were capped and subjected to centrifugation (3220 × *g*, 15 min). The vials were then uncapped, and the hydrated NaCl particles were allowed to air-dry (RT, ∼1 h) before being vacuum-dried (RT, ON, 30 in. Hg), forming a fused salt template for scaffold fabrication.

Precursor solutions of PCL (100% PCL-DA) and a designated PCL/CS-*graft*-PCL (90:10 or 75:25 wt %) were prepared in DCM (0.15 g mL^–1^). A photoinitiator (10 wt % DMP in NVP) was then added at 15 vol % and mixed atop a shaker plate. A designated precursor solution (∼5 mL) was added to a fused salt template, and the vial was sequentially capped, centrifuged (1260 × *g*, 10 min), and exposed to UV light (UV-Transilluminator, 6 mW cm^–2^, 365 nm) for ∼6 min. After cross-linking, vials were uncapped and allowed to air-dry (RT, ∼1 h) in a fume hood before being vacuum-dried (RT, ∼12 h, 30 in. Hg). Salt templates were removed by placing vials in a solution containing water and ethanol (1:1 by vol) for ∼5 days with daily solution changes. The resulting porous scaffolds were air-dried (RT, ∼4 h), vacuum-dried (RT, ON, 30 in. Hg), and annealed under vacuum (85 °C, 1 h, 30 in. Hg). The resulting cylindrical specimen was sliced (*t* ∼ 2 mm) into discs (Vibratome, Leica VT 1000 S) and then biopsy-punched (Integra Miltex, *d* ∼ 6 mm) to produce scaffold specimen discs (*d* ∼ 6 mm × *t* ∼ 2 mm) for analyses.

### 2.5 Film Fabrication

Solid films were prepared via solvent casting in a process analogous to that described above. Precursor solutions were prepared (0.20 g mL^–1^ DCM) and combined with the aforementioned photoinitiator (at 15 vol %). Approximately 2.5 mL of each precursor solution was injected into circular silicone molds (McMaster-Carr, *d* ∼ 45 mm × *t* ∼ 2 mm) secured between two glass slides. Molds were then exposed to UV light for ∼3 min on each side. Swollen films were carefully removed from the molds and air-dried (RT, ON) inside a fume hood. Films were then soaked in an ethanol bath atop a shaker plate (150 rpm, ∼4 h) before being sequentially air-dried (RT, ∼2 h), vacuum-dried (RT, ∼4 h, 30 in. Hg), and annealed under vacuum (85 °C, 1 h, 30 in. Hg). A biopsy punch was used to obtain film specimens (*d* ∼ 6 mm × *t* ∼ 2 mm).

### 2.6 Characterization of Scaffolds and Films

#### 2.6.1 Sol Content

Scaffolds or films (*n* = 3 for each specimen type) were individually submerged in 10 mL of DCM inside sealed 20 mL scintillation vials atop a shaker plate (48 h, 150 rpm). Specimens were removed from vials, air-dried (RT, ON), and finally vacuum-dried (RT, ON, 30 in. Hg). The initial and final masses were used to determine the sol content (i.e., % mass loss).

#### 2.6.2 Thermogravimetric Analysis (TGA)

TGA (Q50, TA Instruments) was performed on scaffolds (*n* = 3) from RT to 600 °C utilizing platinum pans with a heating rate of 10 °C min^–1^ under N_2_.

#### 2.6.3 Scaffold Morphology: Pore Size, Porosity (%), and Pore Interconnectivity (%)

Pore size was evaluated via scanning electron microscopy (SEM, Tescan Vega 3, 10 kV accelerating voltage) images of scaffold specimens (*n* = 5). Cross sections of scaffolds were coated with ∼10 nm of Au–Pt under a vacuum via a Cressington 108 sputter coater. The average pore size was determined from measurements (*n* = 5 from each SEM image; *n* = 25 total measurements) of pores along the diagonal midline utilizing ImageJ software.

The % porosity of scaffolds (*n* = 3) was gravimetrically determined utilizing density measurements of solid films (ρ_solid film_) and corresponding porous scaffolds (ρ_*porous scaffold*_) per eq 1:

$$\mathrm{Porosity}\;(\%)=\frac{\rho_{\mathrm{solid}\mathrm{film}}-\rho_{\mathrm{porous}\mathrm{scaffold}}}{\rho_{\mathrm{solid}\mathrm{film}}}\times 100$$ 1

Scaffold % pore interconnectivity was quantified using a previously established water wicking test.^36^ In short, to expel any air bubbles within pores, scaffolds (*n* = 3) were individually submerged in 10 mL of DI water inside sealed 20 mL scintillation vials atop a shaker plate (24 h, 150 rpm). After removal, scaffolds were weighed (mass_total_), blotted dry with a Kimwipe (to remove DI water within the interconnected pores), and weighed once again (mass_interconnected_). The % pore interconnectivity of scaffolds was calculated per eq 2:

$$\mathrm{Pore Interconnectivity}\;(\%)=\frac{{\mathrm{mass}}_{\mathrm{total}}-{\mathrm{mass}}_{\mathrm{interconnected}}}{{\mathrm{mass}}_{\mathrm{total}}}\times 100$$ 2

#### 2.6.4 PCL *T*_m_ and Crystallinity (%)

Differential scanning calorimetry (DSC, Q100, TA Instruments) was used to determine the *T*_m_ (onset and midpoint) and % crystallinity of PCL within scaffolds (*n* = 3). Scaffold portions (∼10 mg) were hermetically sealed before being heated/cooled (5 °C min^–1^) from 0 to 80 °C over two cycles. Reported values were obtained using the second heating cycle to remove thermal history. The *T*_m_ (midpoint) was determined from the maximum point on the endothermic melt peak for PCL, while % crystallinity was determined per eq 3:

$$\%X_{c}=\frac{\Delta H_{m}}{{{\Delta H}^{{}^{\circ}}}_{c}\times w}\times 100$$ 3

where Δ*H*_m_ is the enthalpy of fusion calculated from the integral of the endothermic melt peak, Δ*H*°_c_ is the enthalpy of fusion for 100% crystalline PCL (139.5 J g^–1^),^37^ and *w* is the mass fraction of the respective polymer (i.e., the portion of the scaffold that is PCL, per Table S1).

#### 2.6.5 Shape Memory: “Self-Fitting” Behavior

The “self-fitting” behavior of scaffolds (*n* = 3) was evaluated using a model defect similar to our previous report.^15^ A drill press (Grizzly G7948) was used to create a circular (*d* = 5 mm) defect from a sheet of ultrahigh-molecular-weight polyethylene (UHMWPE, McMaster-Carr, *t* = 2 mm). Initial scaffold diameters were recorded, and the specimens were subsequently submerged (∼2 min) in a preheated (*T* = 55 °C) water bath atop a hot plate equipped with a digital temperature probe (MR Hei-Tec, Heidolph Scientific). The resulting softened scaffolds were then press-fit into the model defect and allowed to cool to RT (∼3 min). Next, scaffolds were carefully removed from the model defect and allowed to sit for ∼2 min at RT before the “fixed shape” diameters were recorded. Scaffolds were then resubmerged in the heated water bath for ∼2 min before removal and a subsequent waiting period (∼2 min). Finally, the “recovered shape” diameter was recorded. These steps were completed once more to determine shape fixity (*R*_*f*_) and shape recovery (*R*_*r*_) over the first (*N* = 1) and second (*N* = 2) cycle, per eq 4 and eq 5, respectively:

$$R_{f}(N)=\frac{\varepsilon_{u}(N)}{\varepsilon_{m}}\times 100$$ 4

$$R_{r}(N)=\frac{\varepsilon_{i}(N)}{\varepsilon_{r}(N)}\times 100$$ 5

where ε_*u*_(*N*) is the scaffold diameter after being press-fit into the mold, ε_*m*_ is the diameter of the mold, ε_*i*_(*N*) is the diameter of the scaffolds after the shape is recovered, and ε_*r*_(*N*) is the initial diameter of the scaffold. All diameters were measured with electronic calipers (10 μm resolution).

#### 2.6.6 Compressive Mechanical Properties

Scaffolds (*n* = 5) were subjected to static compression testing (Instron 5944, equipped with a 2 kN load cell) at RT until 85% strain. A constant strain rate of 1.5 mm min^–1^ was used for each individual test. The compressive modulus (*E*), compressive strength, and toughness were reported based on the resulting stress–strain curves. The slope of the initial linear region (≤10% strain) was used to determine *E*. Compressive strength was determined from the stress under 85% strain. Toughness was calculated from the integration of the stress–strain curves up to 85% strain. A custom MATLAB code was used to calculate the values for *E*, compressive strength, and toughness.

#### 2.6.7 Contact Angle Analysis

Surface hydrophilicity of films (*n* = 3) was characterized via static water contact angle (θ_static_) measurements using an Attension Theta Flex optical tensiometer (Biolin Scientific) equipped with an autodispersion, video camera, and drop-shape analysis software (OneAttension). A sessile droplet (5 μL) of DI water was placed on the surface of each film, and θ_static_ was measured after 2 min. The reported values are the average and standard deviation of three measurements made on different regions of each of the three films of a given formulation.

#### 2.6.8 Swelling Ratio

Scaffolds or films (*n* = 3) were individually submerged in 10 mL of PBS inside sealed 20 mL scintillation vials atop a shaker plate (RT, 7 days, 150 rpm). Specimens were removed from vials and lightly blotted with a Kimwipe to remove excess PBS. The initial (i.e., dry) and final (i.e., swollen) masses were used to determine swelling ratios (i.e., % water uptake), per eq 6:

$$\mathrm{Swelling Ratio}\;(\%)=\frac{{\mathrm{mass}}_{\mathrm{swollen}}-{\mathrm{mass}}_{\mathrm{dry}}}{{\mathrm{mass}}_{\mathrm{dry}}}\times 100$$ 6

#### 2.6.9 Phase Separation

SEM images of film cross sections were analyzed to assess differences in phase separation (i.e., miscibility).

#### 2.6.10 Degradation

Degradation studies were performed under accelerated conditions, according to ASTM F1635. Scaffolds (*n* = 3 per time point) were individually submerged in 10 mL of 0.05 M NaOH inside sealed 20 mL scintillation vials stored in a 37 °C incubator (VWR Benchtop Shaking Incubator, Model 1570, 60 rpm). At specified time points (up to 21 days), specimens were removed, gently rinsed with DI water, and vacuum-dried (RT, ON, 30 in. Hg). The final dry masses were compared to the initial dry masses to determine scaffold degradation (i.e., % mass loss) at each time point. Scaffolds were not used for subsequent time points.

#### 2.6.11 Biofilm Growth and Retention

The opportunistic fungal pathogen *Candida albicans* (ATCC 10231) was purchased from the American Type Culture Collection (ATCC, Manassas, VA, USA). Biofilm growth and retention were characterized using a previously reported semiautomated, multiwell plate screening methodology, with slight modifications.^38–40^ Solid films (*l* ∼ 100 mm × *w* ∼ 100 mm × *t* ∼ 1.5 mm) were prepared similar to those previously described, punched into circular disc specimens (*d* ∼ 15 mm), and finally adhered to the bottom of 24-well plates in triplicate (*n* = 3) with a silicone adhesive (∼10 μL). Overnight cultures of *C. albicans* were prepared as described previously^41^ and used to assess biofilm development, using both direct contact (i.e., biofilm development on films) or indirect contact (i.e., biofilm development from growth media preconditioned with films) as detailed below.

For direct contact cultures, 1.0 mL aliquots of *C. albicans* suspensions were added to films prepared in 24-well plates and incubated statically at 37 °C for 24 h to allow for cell attachment and biofilm development. After incubation, films were rinsed 3× with 1.0 mL of PBS, and the retained surface-attached biomass was quantified by ATP bioluminescence using a Water-Glo microbial water testing kit (Promega, Madison, WI, USA) by first adding 0.5 mL of lysis reagent to each film and shaking in the dark for 10 min at 150 rpm. Then, 100 μL of the resulting lysis solution was transferred to a 96-well white-walled plate to which 100 μL of detection reagent was added and subsequently mixed by gently swirling for 20 s before measuring luminescence at a 50 ms integration time. Replicates were normalized to an assay control (i.e., growth media without microorganism) and averaged to the films prepared from only PCL (i.e., growth on PCL is considered to be 100%).

For indirect contact cultures, 1.0 mL of biofilm growth media (without *C. albicans*) was added to pristine films prepared in 24-well plates placed atop an orbital shaker (150 rpm) for 24 h at RT. The resulting 1.0 mL leachates/extract-containing growth media were collected and inoculated with a 50 μL suspension of 10^7^ cells/mL in biofilm growth media. A 200 μL aliquot of the inoculated leachates was transferred in triplicate (*n* = 3) to a 96-well polystyrene plate and incubated statically for 24 h at 37 °C. After incubation, the wells were rinsed 3× with PBS, dried for ∼1 h at RT, and stained with 100 μL of a crystal violet powder (CV, VWR, Chicago, IL, USA) solution (0.35% in DI water) for 15 min. The excess CV solution was removed by rinsing three times with DI water and then allowed to dry for ∼1 h at RT. Finally, 150 μL of 33% glacial acetic acid (VWR) was introduced to solubilize CV dye bound to the retained biofilms, and the absorbance was subsequently measured at 600 nm. The resulting data were likewise normalized to a control assay and compared directly to the analogous PCL control.

### 2.7 Statistical Analysis

Quantitative data were obtained using, at minimum, triplicates (*n* ≥ 3), and the results are expressed as the mean ± standard deviation. Statistical analysis was performed using GraphPad Prism 10 (GraphPad Software, Inc., San Diego CA, USA) via one-way analysis of variance (ANOVA) tests, followed by Tukey’s HSD *post hoc* test for multiple comparisons. Statistical significance was considered for *p*-values < 0.05.

## 3 Results and Discussion

### 3.1 CS-*graft*-PCL Synthesis and Characterization

CS-*graft*-PCL copolymers were synthesized using an established procedure, and our observations (e.g., CS-dependent yield and solubility) were consistent with prior reports.^29^ Herein, four CS-*graft*-PCL copolymers were prepared by varying FMRs of glucosamine units in CS to ε-caprolactone (1:6, 1:12, 1:24, and 1:48) (Figure 2a and Table 1). With a change in FMR from 1:6 (highest CS content, ∼28 wt %) to 1:48 (lowest CS content, ∼8 wt %), the yield of CS-*graft*-PCL copolymers increased from ∼54% to ∼91%, respectively. Due to the presence of grafted PCL chains, all CS-*graft*-PCL copolymers showed good solubility in common organic solvents (e.g., DCM and THF), facilitating the formation of scaffolds via SCPL. Below 1:6 FMR (i.e., CS content > ∼28 w%), the copolymer yield (<40%) and solubility (insoluble in DCM) decreased precipitously. Based on ^1^H NMR, the structure of CS-*graft*-PCL copolymers was verified due to the presence of characteristic CS (3.4–3.9, 3.2, and 2.05 ppm) and PCL (4.1, 2.3, 1.6, and 1.4 ppm) peaks (Figure S1). ATR-FTIR further confirmed their chemical composition, namely, due to the presence of absorbance bands at 1734 cm^–1^ and 1660 cm^–1^, which were assigned to the characteristic bands of carbonyl groups of PCL and amide I band of CS, respectively (Figure S2). The average molecular weights of the CS-*graft*-PCL copolymers were determined via GPC (Table 1 and Figure S3). Average *M*_w_ values ranged from ∼27 to ∼34 kg mol^–1^ and decreased with greater PCL content (i.e., highest *M*_w_ observed for 1:6). This trend could potentially be explained by an increase in steric hindrance that limits graft PCL chain growth. The wt % CS comprising each CS-*graft*-PCL copolymer was determined via TGA (Figure S4): 1:6 (∼28%), 1:12 (∼17%), 1:24 (∼10%), and 1:48 (∼8%) (Table 1).

### 3.2 Scaffold and Film Fabrication

PCL/CS-*graft*-PCL semi-IPN scaffolds and a PCL network scaffold control (i.e., 100% PCL-DA) were successfully fabricated via an established SCPL protocol (Figure 2b). Analogous films were also prepared via solvent casting for certain analyses. A total of eight PCL/CS-*graft*-PCL compositions were prepared. Composition designations are based on both PCL-DA to CS-*graft*-PCL wt % ratios (i.e., 90:10 or 75:25) and the reaction FMR (i.e., glucosamine units in CS to ε-caprolactone) of the CS-*graft*-PCL (1:48, 1:24, 1:12, or 1:6) (Figure 2c). For example, those formed from a 75:25 wt % ratio of PCL-DA to CS-*graft*-PCL (1:12) are denoted as “*75:25/1:12*”. The varying amounts of CS in the CS-*graft*-PCL copolymers led to differences in CS content of the scaffolds from ∼0.8 wt % (for *90:10/1:48*) to ∼7 wt % (for *75:25/1:6*) (Figure 2c and Table S1).

Sol content values (i.e., levels of non-cross-linked materials) were determined to confirm adequate cross-linking of PCL-DA upon the formation of PCL/CS-*graft*-PCL semi-IPN scaffolds and films (Figure S5 and Table S2). PCL-only (i.e., no CS-*graft*-PCL) scaffolds and films had minimal sol content values of ∼2.4% and ∼3.3%, respectively, confirming sufficient cross-linking of PCL-DA macromers (i.e., > 95% cross-linking). For semi-IPN compositions, sol content values were expected to align with the level of non-cross-linkable CS-*graft*-PCL. In the case of 90:10 compositions, scaffolds did not exceed ∼12% sol content, while films did not exceed ∼14%. Likewise, 75:25 compositions displayed sol contents of ∼25% and ∼29% for scaffolds and films, respectively. These results confirm that PCL-DA (>90% acrylation) was able to undergo cross-linking in the presence of non-cross-linkable CS-*graft*-PCL, effectively forming the designated PCL/CS-*graft*-PCL semi-IPN compositions with targeted values of the graft copolymer (i.e., either ∼10% or ∼25%).

As mentioned above, achievement of the targeted wt % ratios of PCL-DA to CS-*graft*-PCL (90:10 or 75:25) was confirmed through TGA of scaffolds (Figure S6). The CS-*graft*-PCL copolymers (Figure S4a) underwent a distinct onset of thermal degradation at a temperature lower (∼250–300 °C) than that of the cross-linked PCL networks (>400 °C). Indeed, the mass loss below 400 °C was ∼10% for the 90:10 compositions and ∼25% for the 75:25 compositions, confirming the targeted level of CS-*graft*-PCL comprising the semi-IPN scaffolds.

### 3.3 Scaffold Morphology

Scaffolds were readily prepared via SCPL from precursor solutions containing CS (i.e., PCL-DA and PCL/CS-*graft*-PCL) or only PCL (i.e., 100% PCL-DA). SEM images of scaffold cross sections confirmed the presence of interconnected macropores, achieved through the use of fused salt templates (Figure 3). Further analysis of scaffold SEM images revealed that the average pore size for all compositions was similar at ∼240 μm (Figure S7a and Table S3), well within the range associated with osteogenesis and neotissue infiltration, respectively.^42,43^ Regardless of CS content, scaffolds were also highly porous (∼60% porosity) (Figure S7b and Table S3) and exhibited good poor interconnectivity (∼45–55%) (Figure S7c and Table S3).

**Figure 3:**
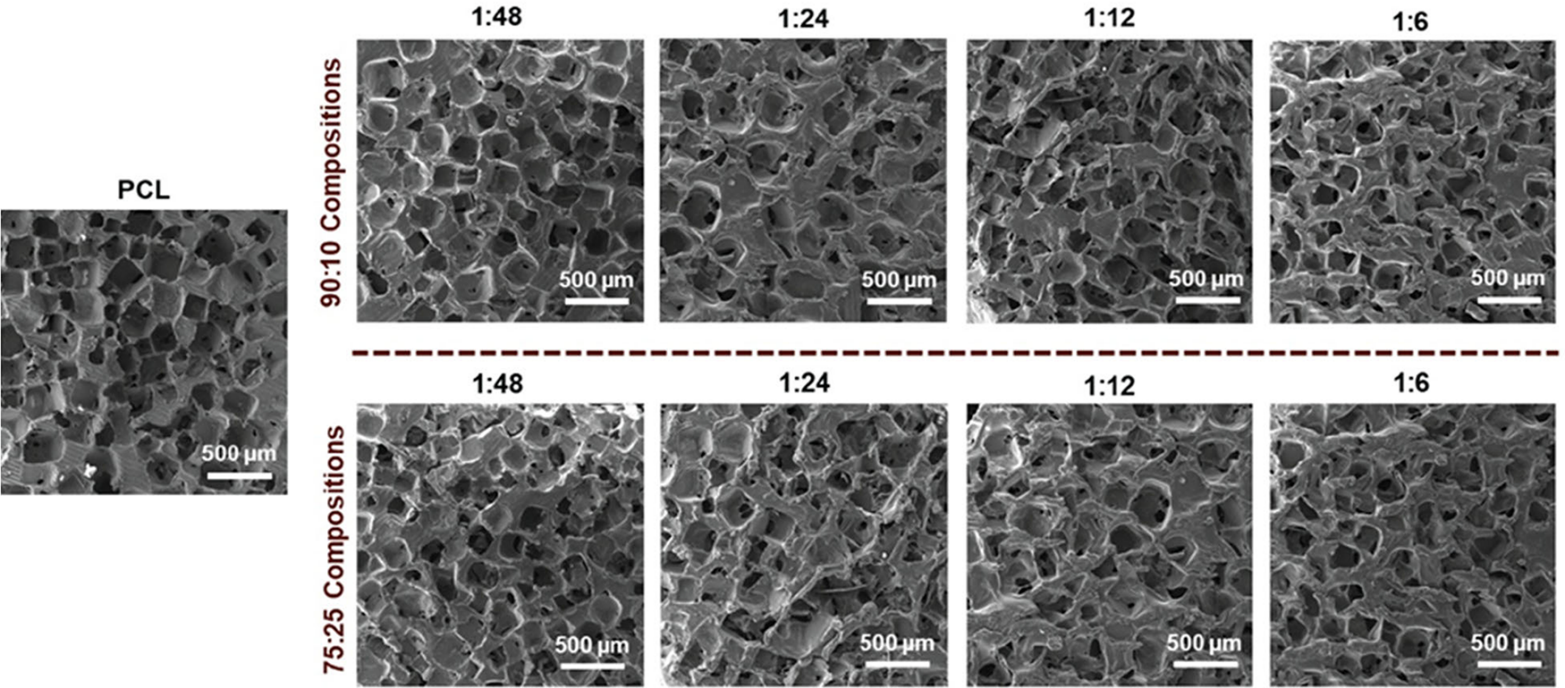
SEM images of scaffold cross sections.

### 3.4 PCL *T*_m_ and Crystallinity (%)

Scaffold “self-fitting” behavior occurs at *T*_m_ of PCL (∼55 °C), which represents the transition temperature (i.e., *T*_trans_) at which the scaffold must be heated for shape recovery within the defect and then cooled below for shape fixation. Beyond serving as switching segments for shape memory behavior, the PCL crystalline lamellae have significant impact on both mechanical and degradative properties.^16^ Therefore, the *T*_m_ and % crystallinity of PCL within the eight PCL/CS-*graft*-PCL semi-IPN scaffolds were quantified via DSC and compared to those of the PCL scaffold control (Figure S8 and Table S4). While there was no significant impact on the PCL *T*_m_ (∼52–54 °C) (Figure S8b), there was a reduction in PCL % crystallinity for all PCL/CS-*graft*-PCL scaffolds (∼32–36%) versus the PCL control (∼43%) (Figure S8c). Although a decrease in PCL crystallinity is expected to give rise to lower *T*_m_ values, these results indicate that the amount of CS (∼0.8 to ∼7 wt %) within semi-IPN scaffolds was insufficient to suppress the PCL *T*_m_ (Table S1). This agrees with Senda et al.,^44^ who reported that for PCL/CS physical blends, the PCL *T*_m_ was not decreased until the CS content exceeded 20%, while PCL % crystallinity decreased significantly as CS wt % was increased. For PCL/CS-*graft*-PCL scaffolds, the reduction of PCL crystallinity with these relatively low amounts of CS may have resulted from the formation of a miscible phase between PCL and CS (per Section 3.7, Figure S12), primarily due to intermolecular hydrogen bonding between the carbonyl groups of PCL and the hydroxyl/amine groups present in CS. In addition, the reduction in PCL % crystallinity could be due to differences in molecular mobility between PCL and CS. For instance, since the crystallization temperature of CS is higher than the glass transition temperature ( *T*_g_) of PCL, the PCL molecules in the amorphous phase may be trapped in this glassy environment (i.e., at RT, the amorphous region of CS exists in the glassy state) if PCL and CS are miscible in the amorphous phase.^44,45^

### 3.5 “Self-Fitting” Behavior

Scaffold specimens (*d* ∼ 6 mm) were quantitatively evaluated for their “self-fitting” behavior based on previously developed methods.^15^ The circular (*d* ∼ 5 mm) model defect employed for testing is representative of the bilateral rat calvarial defect model often utilized for preliminary bone healing studies.^46,47^ Scaffolds were first exposed to warm water (i.e., *T* = 55 °C) before being press-fit into the model defect and allowed to cool to RT. After removal from the model defect, the ability to maintain this new “fixed shape” (i.e., *R*_*f*_) was assessed for each scaffold. Scaffolds were then resubmerged in warm water and allowed to expand to their final “recovered shape” to quantify the shape recovery (i.e., *R*_*r*_). As noted above, the PCL crystalline lamellae serve as switching segments for shape memory behavior. Despite a reduction in PCL crystallinity for these eight PCL/CS-*graft*-PCL scaffolds versus the PCL scaffold control, *R*_*f*_ and *R*_*r*_ over both cycles remained near ∼100% (Table S5), indicating that PCL crystallinity (∼32–36%) was sufficient. Moreover, given the high degree of cross-linking (>95%) within PCL-DA networks (per Section 3.2), the effects of reduced crystallinity within CS-containing composites may have been minimized. Furthermore, this process demonstrates the favorable handleability of such scaffolds during the self-fitting process.

### 3.6 Compressive Mechanical Properties

For bone regeneration of CMF defects, it is essential that PCL/CS-*graft*-PCL scaffolds retain the favorable mechanical properties of PCL scaffolds. Pristine CS scaffolds are known to exhibit poor mechanical robustness (e.g., low strength),^48^ limiting their utility. Thus, the presence of CS and the observed reduction in PCL crystallinity have the potential to diminish the mechanical properties of PCL/CS-*graft*-PCL scaffolds. Static compression testing was performed to determine scaffold *E*, strength, and toughness (Figure S9 and Table S6). The compressive *E* values were not statistically different versus that of the PCL scaffold control (∼6.1 MPa), remaining within the range of trabecular bone.^49^ Furthermore, there was no significant reduction in compressive strength (∼31 MPa) or toughness (∼5.3 MJ m^–3^) versus the PCL scaffold control. Importantly, nonbrittle behavior necessary to withstand postsurgical fracture was likewise observed for all PCL/CS-*graft*-PCL scaffolds (i.e., no fracture when tested up to 85% strain). The retention of mechanical robustness is attributed to the semi-IPN design, which maintains a cross-linked PCL network, and to the inclusion of CS at modest levels (∼0.8 to ∼7 wt %) as CS-*graft*-PCL copolymers, which in turn maintain adequate PCL crystallinity (∼32–36%).

### 3.7 Hydrophilicity and Miscibility

Prior to evaluating the in vitro degradation behavior of PCL/CS-*graft*-PCL semi-IPN scaffolds, surface hydrophilicity and swelling (by water), as well as miscibility (i.e., phase separation), were assessed. CS is a hydrophilic polymer, attributed to its polar functional groups, and has been blended with PCL in various forms (e.g., nanofibers and thin films) to enhance its surface properties (i.e., hydrophilicity).^50,51^ Herein, surface hydrophilicity (i.e., θ_static_) was determined for solid films of compositions analogous to those of scaffolds. Compared with the PCL control (∼90°), statistically lower θ_static_ values were observed for compositions with CS contents ≥ 2.5 wt % (Figure S10 and Table S7). θ_static_ values generally decreased with increasing CS wt % from ∼75° for those containing 2.5 wt % [*75:25/1:24*] and 2.8 wt % [*90:10/1:6*] to ∼70° for those containing 4.4 wt % [*75:25/1:12*] and 7.1 wt % [*75:25/1:6*].

To further assess hydrophilicity, swelling ratios (i.e., water uptake) were determined for PCL/CS-*graft*-PCL scaffolds and films after 7 days in PBS (Figure S11 and Table S8). The PCL scaffold control, owing to its hydrophobicity (θ_static_ ∼90°), exhibited a swelling ratio of just ∼70%. All 75:25 scaffold compositions (∼2–7 wt % CS) had swelling ratios significantly higher, ranging from ∼158% to ∼176%. Overall, 90:10 scaffold compositions (∼0.8–2.8 wt % CS) absorbed less water than the 75:25 scaffolds, although the difference was not statistically significant. The *90:10/1:12* scaffold displayed a significantly higher swelling ratio (∼147%) compared to the PCL control. Overall, these results suggest that PCL/CS-*graft*-PCL scaffolds with sufficient CS contents can increase water uptake. Perhaps due to the lower surface areas versus scaffolds, the films exhibited no differences among compositions under these testing conditions.

We have previously reported that for PCL-based semi-IPNs (e.g., PCL/PLLA semi-IPNs), compositions that produced partial miscibility led to accelerated rates of degradation relative to neat PCL due to their increased water uptake.^52^ In contrast, highly immiscible compositions were degraded relatively slowly. Herein, SEM images were utilized to determine the extent of phase separation (i.e., immiscibility) within films (Figure S12). SEM analysis has previously been used for blended polymer systems, where phase separation is marked by varying extents of coalescence, or defined circular regions, where one polymer is separated from the other.^53,54^ In line with our previous report,^52^ pristine PCL films exhibited the highest degree of miscibility, evidenced by their uniform morphologies and contributed to their slow degradation rates. For PCL/CS-*graft*-PCL films, immiscibility qualitatively appeared to increase with increased CS content; however, all compositions were deemed to be partially miscible rather than highly immiscible. The partial miscibility of these compositions was expected to lead to faster biodegradation due to an increase in water penetration.

### 3.8 Degradation Studies

Scaffolds with robust degradation rates that correspond to the formation of this new tissue are essential for osteoinductivity and CMF bone defect healing.^8,55^ PCL is known to degrade slowly (∼2 years), limiting the efficacy of PCL scaffolds in bone regeneration by inhibiting neotissue infiltration, which occurs over the course of just ∼2–3 months.^56,57^ With the observed reduction of PCL crystallinity, as well as enhanced hydrophilicity and phase separation, PCL/CS-*graft*-PCL semi-IPN scaffolds were expected to produce increased rates of hydrolytic degradation. This was assessed under accelerated conditions (0.05 M NaOH, 37 °C, 60 rpm; Figure 4 and Table S9). Degradation rates were largely dependent on the wt % CS present in the scaffold. The *90:10/1:48* scaffold, with the lowest CS content (∼0.8 wt %), degraded the slowest, similar to the PCL scaffold control. For the remaining 90:10 scaffolds, as CS content increased from ∼1 wt % (*90:10/1:24*) to ∼2.8 wt % (*90:10/1:6*), degradation rates generally progressively increased. This trend was likewise observed for the 75:25 scaffolds. The fastest degrading scaffolds were those with higher CS contents: ∼2.5 wt % (*75:25/1:24*), ∼4.4 wt % (*75:25/1:12*), and 7.1 wt % (*75:25/1:6*), and correspond to scaffolds with the highest degree of swelling and films with the lowest θ_static_. Given that CS and PCL are biodegradable, PCL/CS-*graft*-PCL semi-IPN scaffolds are expected to show similar trends regarding their in vivo degradation rates (i.e., biodegradation rates dependent on wt % CS comprising each scaffold). Furthermore, the hydrolysis of PCL ester bonds is expected to lead to the cleavage of attached acrylate groups, which, in turn, are metabolized and excreted from the body through urine and CO_2_.

**Figure 4:**
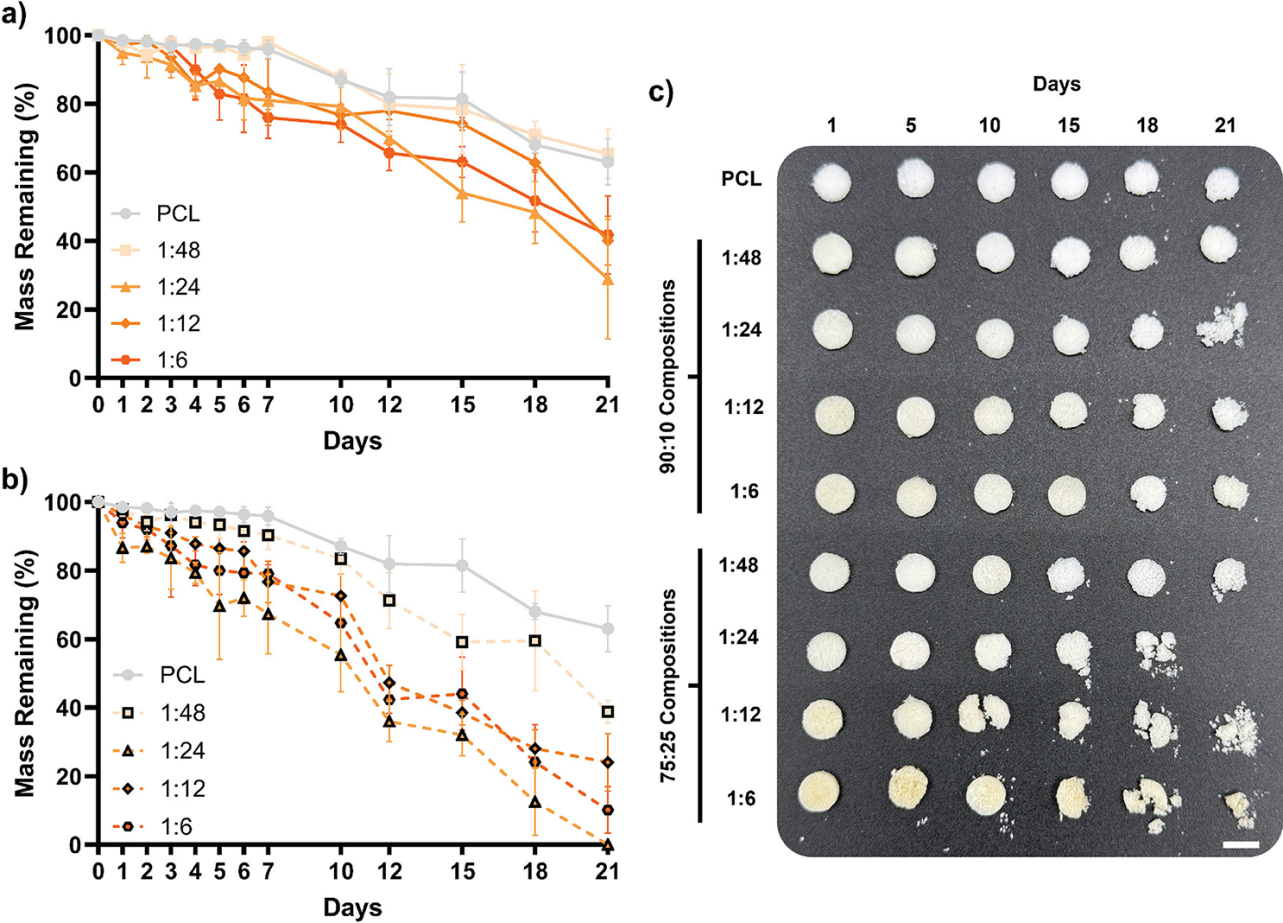
Scaffold gravimetric mass loss over time under accelerated conditions (0.05 M NaOH, 37 °C, and 60 rpm). (a) 90:10 compositions and (b) 75:25 compositions mass loss over 21 days compared to PCL. (c) Representative photos of scaffold specimens at different time points during degradation study (scale bar = 6 mm).

### 3.9 Biofilm Growth and Retention

To reduce the potential for implant-associated infections, a scaffold should prevent the growth and retention of biofilms on its surface.^18^ While PCL scaffolds lack innate antimicrobial behavior, the broad-spectrum antimicrobial activity of CS^23,24^ has potential to be imparted to PCL/CS-*graft*-PCL scaffolds. Considering the prevalence of the fungus *C. albicans* in medical device-related infections,^17,58^ it was selected for biofilm assays. In addition to being notoriously difficult to eradicate, biofilms produced by *C. albicans* have been shown to mature faster than those produced by *Staphylococcus aureus* (*S. aureus*) while often growing larger and more complex than other *Candida* species,^59–61^ further promoting its selection for this study. Moreover, it has been suggested that CS may interfere with the *C. albicans* cell wall organization processes, ultimately impairing its integrity (i.e., leading to an increase in biofilm sensitivity to antibiotics).^62^ While CS exhibits minimum inhibitory concentration (MIC) values that vary depending on several factors (e.g., target microorganism, CS molecular weight, CS degree of deacetylation, etc.), aqueous CS solutions, even at low concentrations (<0.02 wt %), have been shown to disrupt *C. albicans* biofilms.^63^

For direct contact assays, surfaces were directly exposed to *C. albicans*, and the resulting biofilm quantified after 24 h (Figure 5 and Table S10). Despite having the lowest CS content of ∼0.8 wt %, *90:10/1:48* showed a reduction in biofilm on its surface versus that of the PCL control. However, all other PCL/CS-*graft*-PCL compositions, having ≥∼1 wt % CS (∼1 to ∼7.1 wt %), reduced biofilm development to a greater extent. These results demonstrate that PCL/CS-*graft*-PCL formulations produce surfaces with antimicrobial activity at low levels of CS (≥∼0.8 wt %). It is inferred that scaffold surfaces, upon degrading and CS content being reduced, would retain their antimicrobial activity up to a threshold of ≥∼0.8 wt % CS.

**Figure 5:**
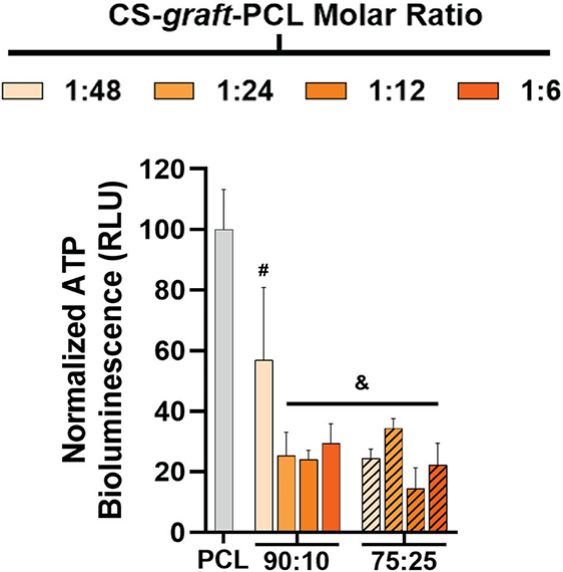
Direct contact assay—*C. albicans* biofilm formation on surfaces of PCL and PCL/CS-*graft*-PCL films (24 h). Data are normalized to biofilm formation on PCL films. Statistical significance is indicated by symbols (where # represents *p* < 0.01 and & represents *p* < 0.0001 relative to growth on PCL).

In addition to mitigating biofilm formation directly onto PCL/CS-*graft*-PCL surfaces, leachates (e.g., water-solubilized CS) that are extracted from the surrounding environment may also contribute to antimicrobial activity. This effect was isolated using indirect assays, wherein films were incubated in growth media (24 h), and the resulting leachate/extract-containing growth media were sequentially collected and inoculated with *C. albicans*, and an aliquot was placed in the well of a polystyrene plate (24 h) (Figure 6 and Table S11). Compared with leachates from the PCL control, those from PCL/CS-*graft*-PCL compositions produced a substantial reduction in biofilm development. The leachate potency was dependent on the nature of the CS-*graft*-PCL copolymers in terms of CS wt % and aqueous solubility (i.e., decreased hydrophobic PCL graft length). A 2-fold biofilm reduction was observed for specimens based on CS-*graft*-PCL (1:48) (i.e., *90:10/1:48* and *75:25/1:48*), having the lowest CS content and highest PCL graft length. The remaining specimens produced an even greater (5-fold) biofilm reduction owing to the higher CS contents and solubilities of the CS-*graft*-PCL (1:24, 1:12, 1:6) copolymers. These trends also generally coincide with greater hydrophilicity (Figure S10), which may facilitate aqueous extraction.

**Figure 6:**
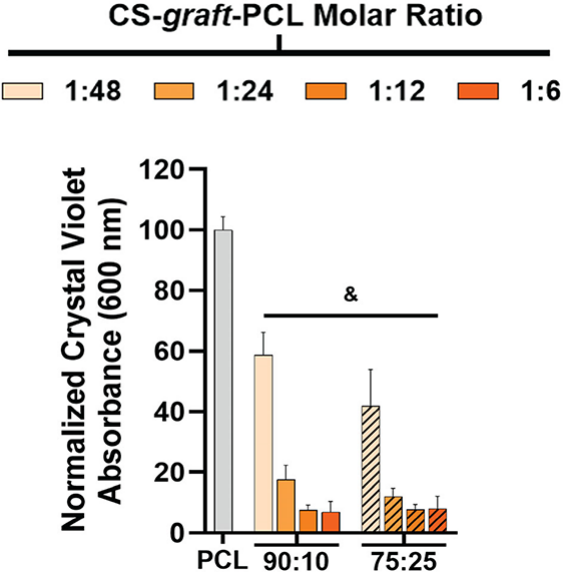
Indirect contact assays—*C. albicans* biofilm formation after incubation of films with growth media (24 h), and the resulting leachate/extract-containing growth media sequentially collected, inoculated, and maintained in the well of a polystyrene plate (24 h). Data are normalized to biofilm formation from PCL films. Statistical significance is indicated by symbols (where & represents *p* < 0.0001 relative to biofilms exposed to PCL leachates).

## 4 Conclusions

Regenerative scaffolds capable of achieving a conformal fit in irregular cranial defects and providing antimicrobial function would be a considerable improvement over the current repair options. Our group has previously developed “self-fitting” SMP bone scaffolds based on thermoresponsive PCL-DA with shape recovery to fill irregular defect geometries. While capable of providing a conformal fit, these PCL-based scaffolds lacked the ability to mitigate infection, which may limit their capacity to act solely in bone tissue repair. Furthermore, PCL degrades extremely slowly (∼2 years) relative to neotissue formation (∼2–3 months). Recently, the use of hybrid polymer systems (e.g., those enriched with natural polymers) has emerged as an effective way to improve the functionality of engineered bone scaffolds. In this work, hybrid (i.e., formed from synthetic and naturally derived polymer) porous scaffolds with tunable degradation rates, antimicrobial properties, and the capacity to achieve defect-specific geometries via shape memory were described. Eight PCL/CS-*graft*-PCL semi-IPN scaffold compositions were prepared by combining PCL-DA with four distinct CS-*graft*-PCL copolymers (i.e., 1:6, 1:12, 1:24, and 1:48) at two separate wt % ratios (i.e., 90:10 and 75:25). These hybrid scaffolds were directly compared to those containing only PCL (i.e., 100% PCL-DA) to determine the effects of both CS content and PCL grafting ratios on the pertinent properties related to bone regeneration and antimicrobial behavior. Altogether, the results presented in this study provide substantial evidence in support of our hypothesis; the inclusion of CS is sufficient in terms of accelerating the rate of degradation and imparting antimicrobial function to PCL-based SMP scaffolds. Scaffolds were successfully formed with interconnected macropores (∼240 μm), conducive to osteogenesis. Shape memory behavior (i.e., the ability to conformally fit within irregular defects) was preserved due to a sufficient retention of PCL crystallinity in scaffolds containing CS (i.e., PCL/CS-*graft*-PCL). Semi-IPN scaffolds also possessed desirable characteristics for the intended application of CMF bone defect treatment (i.e., were mechanically robust), maintaining compressive *E* values in the range of trabecular bone. Furthermore, scaffolds displayed nonbrittle behavior (i.e., did not fracture when tested up to 85% strain), indicating their suitability for handling and press-fitting. The incorporation of CS (a natural hydrophilic polymer) improved the hydrophilicity of scaffolds and analogous films (i.e., based on swelling and θ_static_); hence, scaffold hydrolytic degradation was also enhanced (when tested under accelerated conditions). Specifically, 75:25 scaffold compositions degraded faster than their respective 90:10 counterparts due to increased swelling (i.e., water penetration). Our *75:25/1:24* scaffolds displayed the fastest degradation, followed by *75:25/1:6* and *75:25/1:12* scaffolds. In addition to their improved hydrophilicity, these compositions were also deemed to be partially miscible under SEM, which we have previously reported to be a determining factor in the degradation behavior of these PCL-based polymer systems. Regarding the antimicrobial behavior of the hybrid composites, the addition of CS imparted significant antimicrobial function relative to the PCL control in both direct and indirect contact cultures. Moreover, it appears that a minimal amount of CS (i.e., ∼0.8 wt %) within the composite system is sufficient to reducing the growth of *C. albicans* biofilm on film surfaces, as observed in our direct contact evaluation. However, bulk CS concentrations may play less of a role when compared with PCL grafting ratios with regard to CS leaching (i.e., the ability of our composites to disrupt adjacent biofilms), which was observed in our indirect contact evaluation. Nevertheless, it appears that the antimicrobial activities observed herein are a result of not only surface interactions between CS and *C. albicans* but also the tendency for CS to solubilize into the aqueous environment. Overall, the scaffolds formed in this study would provide significant improvements to current repair options by reason for improved degradation, innate antimicrobial function, and potentially enhanced osteogenic cellular responses (due to the addition of CS, a natural biopolymer). In future studies of these hybrid composite scaffolds, in vitro cellular mineralization and in vivo bone formation and infection resistance will also be assessed.

## Supplementary Material



## Notes

Detailed methods for CS-*graft*-PCL copolymer synthesis and characterization; ^1^H NMR spectra; ATR-FTIR spectra; GPC chromatographs; TGA thermograms of CS-*graft*-PCL copolymers; CS content (wt %) for scaffolds and films; sol content data; TGA thermograms of PCL/CS-*graft*-PCL scaffolds; scaffold pore properties; DSC thermograms and scaffold thermal properties; scaffold shape memory data; compressive mechanical properties of scaffolds; water contact angle measurements of films; scaffold and film swelling data; SEM images of film cross sections; scaffold accelerated degradation data; normalized ATP bioluminescence values of *C. albicans* biofilm formation on film surfaces; and normalized crystal violet absorbance values of *C. albicans* biofilm growth following incubation in growth media containing film leachates/extracts (PDF)
