# Supplementary material for: Hybrid Chitosan/PCL Shape Memory Scaffolds with Potential for Bone Regeneration and Infection Resistance
Source: ACS Biomater Sci Eng. 2025 Aug 21;11(9):5627–37. doi: 10.1021/acsbiomaterials.5c01160 (PMC12421515; doi:10.1021/acsbiomaterials.5c01160)
Supplement: Supplementary file 1 [file ab5c01160_si_001.pdf]

Supporting Information

Hybrid Chitosan/PCL Shape Memory Scaffolds with Potential for Bone Regeneration and Infection Resistance

Damion T. Dixon[1,], Ainsley G. Shields[1], Shane J. Stafslien[2], Lyndsi Vander Wal[2], and Melissa A. Grunlan[1,3,4,*]*

[1]Department of Biomedical Engineering, Texas A&M University, College Station, Texas 77843, United States

[2]Department of Coatings and Polymeric Materials, North Dakota State University, Fargo, North Dakota 58108, United States

[3]Department of Materials Science and Engineering, Texas A&M University, College Station, Texas 77843, United States

[4]Department of Chemistry, Texas A&M University, College Station, Texas 77843, United States

***Corresponding Authors**:

Damion T. Dixon
Email: dtd@tamu.edu

Melissa A. Grunlan
Email: mgrunlan@tamu.edu

19 Pages

12 Figures

11 Tables

1. SUPPORTING METHODS

1.1 CS-*graft*-PCL Synthesis and Characterization

Four CS-*graft*-PCL copolymers were synthesized by varying the FMR between the glucosamine units in CS and the amount of ε-caprolactone (1:6, 1:12, 1:24, and 1:48). The % degree of deacetylation (DDA) of CS was first determined via ^{1}H-NMR spectroscopy (Avance NEO 400 MHz spectrometer) per a prior report.[1] Approximately 5 mg of dried (50 °C, 30 in. Hg, 24 h) CS was added to a 5 mm NMR tube containing ~500 µL of a 2% deuterium chloride (DCl) solution (in D_2O) and heated (~70 °C) for ~1 h until the CS had completely dissolved. ^{1}H-NMR was recorded at RT with an acquisition time of 1 s and relaxation delay of 12 s. The % DDA was calculated per eq 1:

$$\%\ \mathrm{DDA} = [1 - \frac{\frac{1}{3}\times I_{CH3}}{\frac{1}{6}\times I_{(H2-H6)}}] \times 100 \qquad (1)$$

where I_{CH3} is the integral of *N*-acetyl groups and $I_{(H2\text{-}H6)}$ is the summation of integrals of H_2, H_3, H_4, H_5, and H_6.[2] The calculated % DDA was ~86% (n = 3), equating to 6.0 mmol of glucosamine units per 1.0 g (i.e., the value used to calculate grafting ratios between CS and PCL).

CS-*graft*-PCL copolymers were analyzed via ^{1}H-NMR spectroscopy similar to PCL, using $CDCl_3$ as the standard. Representative ^{1}H-NMR spectra for CS, PCL, and CS-*graft*-PCL are shown in **Figure S1**. The peaks at 3.4–3.9, 3.2, and 2.05 ppm were assigned to $H_{3,4,5,6,6'}$, $H_{2'}$, and H_7 of CS, respectively. The peaks of H_d, H_c, H_b, and H_a in PCL were present at 4.1, 2.3, 1.6, and 1.4 ppm, respectively. The chemical structure of CS-*graft*-PCL was confirmed via its ^{1}H-NMR spectra, due to the presence of characteristic CS and PCL peaks.[3]

ATR-FTIR spectroscopy (Bruker ALPHA-Platinum, n = 32) was performed on CS, PCL, and their copolymers to further confirm their chemical composition. All spectra were recorded in

the range of 4000–500 cm^{-1} and normalized to the same scale for comparison (**Figure S2**). The observed results agreed previous reports for hybrid CS-*graft*-PCL copolymers.[4, 5] In the spectra for CS (**Figure S2a**), the characteristic peak at 890 cm^{-1} was related to the C−O stretching of glycoside and the peak at 1160 cm^{-1} was related to asymmetric C−O stretching. Additionally, the two peaks at 1638 cm^{-1} and 1660 cm^{-1} were attributed to C−N and C=O stretching of amide I bands, respectively. A strong peak at 1734 cm^{-1} was observed in the spectra for PCL (**Figure S2b**), which can be attributed to the C=O stretching of carbonyl groups. The PCL spectra also displayed asymmetric CH_2 stretching vibration and symmetric CH_2 stretching at 2940 cm^{-1} and 2860 cm^{-1}, respectively. All of the CS-*graft*-PCL copolymers presented absorbance bands at 1734 cm^{-1} and 1660 cm^{-1}, which were assigned to the characteristic bands of carbonyl groups of PCL and amide I band of CS, respectively.

The molecular weights of the CS-*graft*-PCL copolymers were determined via GPC (TOSOH ambient temperature GPC, Tosoh Bioscience). Samples were dissolved at 1 mg/mL in tetrahydrofuran (THF), filtered (0.45 µm syringe filter, Sigma-Aldrich), and their molecular weights (**Table 1**) determined utilizing a PS standard. Representative GPC chromatographs are shown in **Figure S3**.

Lastly, the wt% CS comprising each CS-*graft*-PCL copolymer was determined via TGA (Q50, TA Instruments) (**Table 1**). Samples (~10 mg, $n = 3$ for each CS-*graft*-PCL copolymer) were tested from RT to 600 °C utilizing platinum pans with a heating rate of 10 °C min^{-1} under N_2. Representative TGA thermograms and wt% CS (i.e., mass remaining) are shown in **Figure S4**.

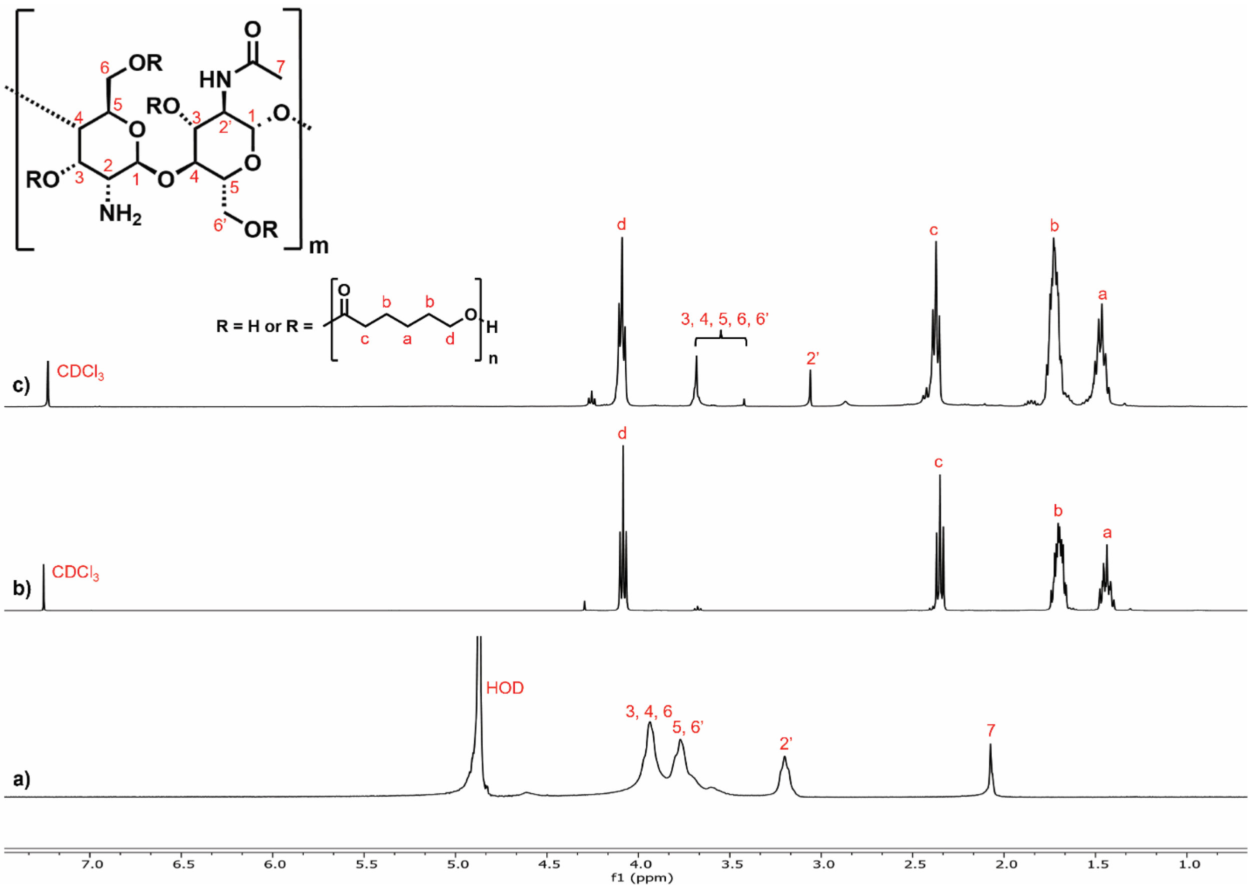

Figure S1. Representative ^{1}H-NMR spectra of (a) CS, (b) PCL, and (c) CS-*graft*-PCL (1:12).

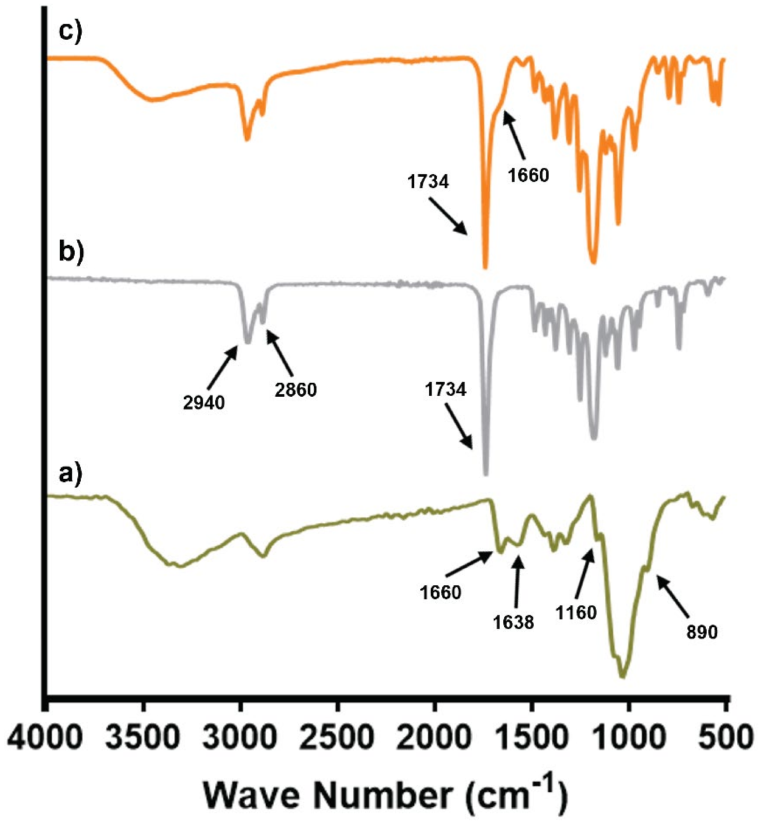

Figure S2. Representative ATR-FTIR spectra of (a) CS, (b) PCL, and (c) CS-*graft*-PCL (1:12).

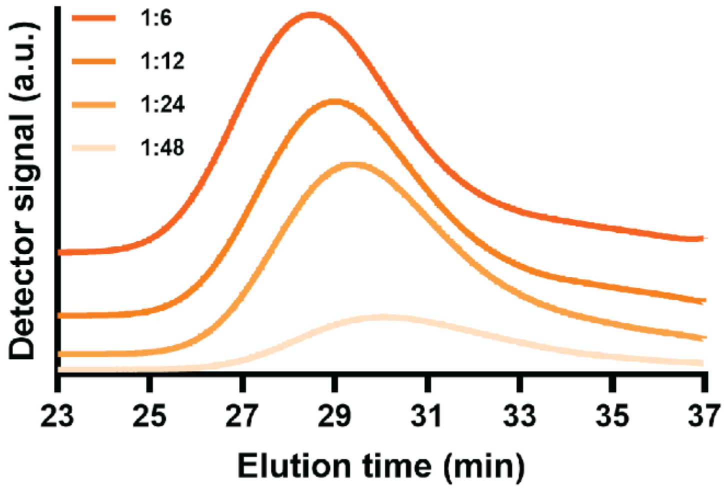

Figure S3. GPC chromatographs of CS-*graft*-PCL copolymers (1:6, 1:12, 1:24, and 1:48).

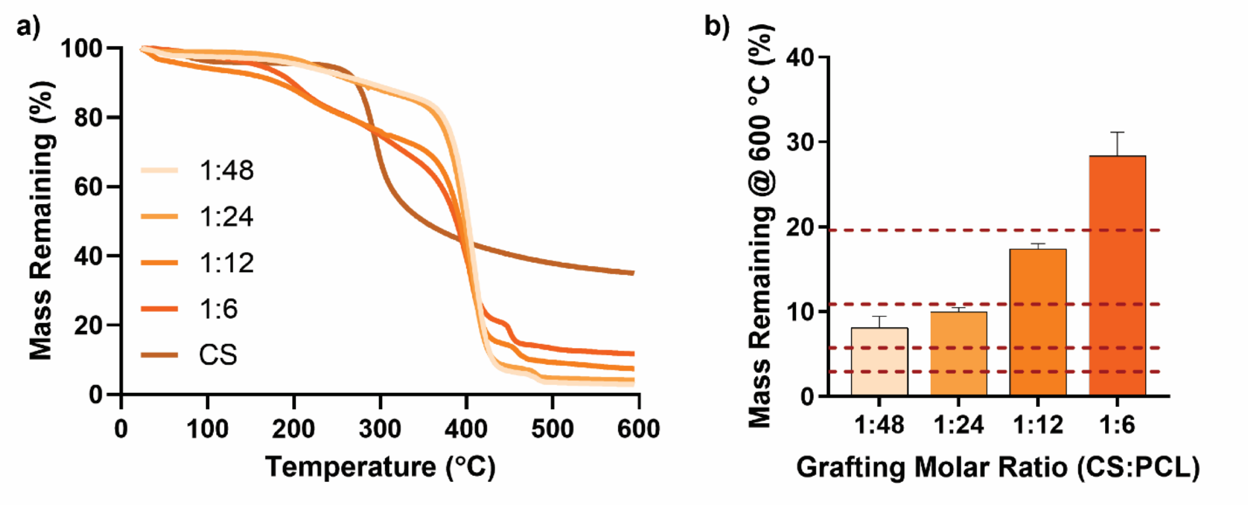

Figure S4. (a) TGA thermograms of chitosan (CS) and CS-*graft*-PCL copolymers (1:6, 1:12, 1:24, and 1:48). (b) wt% plateau values at 600 °C, corresponding to wt% CS comprising each CS-*graft*-PCL copolymer. Plateau values are based on CS % mass remaining at 600 °C; “dashed” lines indicates estimated wt% CS based on copolymer synthesis (i.e., relative wt% of CS added to each reaction).

Table S1. PCL/CS-*graft*-PCL chitosan (CS) wt%.

Sample	CS Content[a] (wt%)
**90:10 Compositions**	
1:48	0.81 ± 0.11
1:24	0.99 ± 0.04
1:12	1.74 ± 0.05
1:6	2.84 ± 0.23
**75:25 Compositions**	
1:48	2.03 ± 0.28
1:24	2.50 ± 0.10
1:12	4.35 ± 0.13
1:6	7.10 ± 0.56

[a]Determined via TGA of scaffolds.

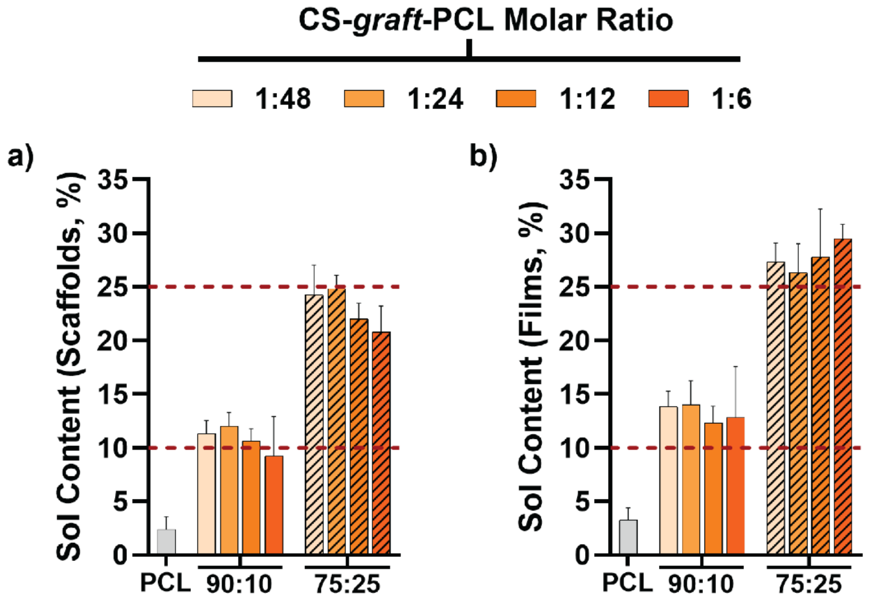

Figure S5. Sol content of (a) scaffolds and (b) films. “dashed" lines (10 or 25%) correspond to wt% non-cross-linkable CS-*graft*-PCL copolymers formulated into scaffolds and films.

Table S2. Sol content values (wt%) of scaffolds and films.

Composition	Sol Content (Scaffolds, %)	Sol Content (Films, %)
PCL	2.42 ± 1.16	3.28 ± 1.12
**90:10 Compositions**		
1:48	11.31 ± 1.22	13.84 ± 1.42
1:24	12.03 ± 1.26	14.01 ± 2.21
1:12	10.62 ± 1.16	12.32 ± 1.53
1:6	9.22 ± 3.68	12.83 ± 4.74
**75:25 Compositions**		
1:48	24.25 ± 2.78	27.33 ± 1.74
1:24	24.84 ± 1.23	26.33 ± 2.68
1:12	22.00 ± 1.48	27.79 ± 4.44
1:6	20.79 ± 2.42	29.51 ± 1.32

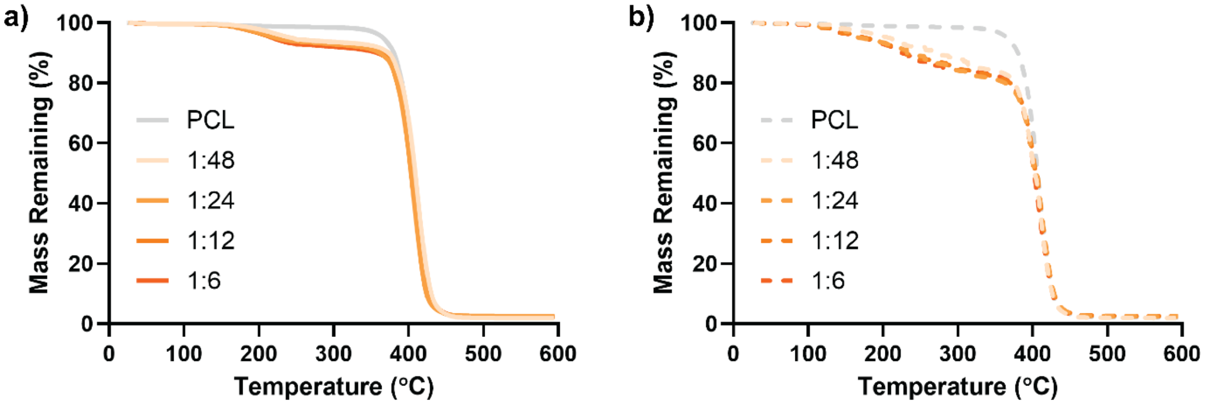

Figure S6. TGA thermograms of (a) 90:10 composition scaffolds and (b) 75:25 composition scaffolds confirming relative (i.e., ~10% or ~25%, respectively) wt% of non-cross-linkable CS-*graft*-PCL copolymers.

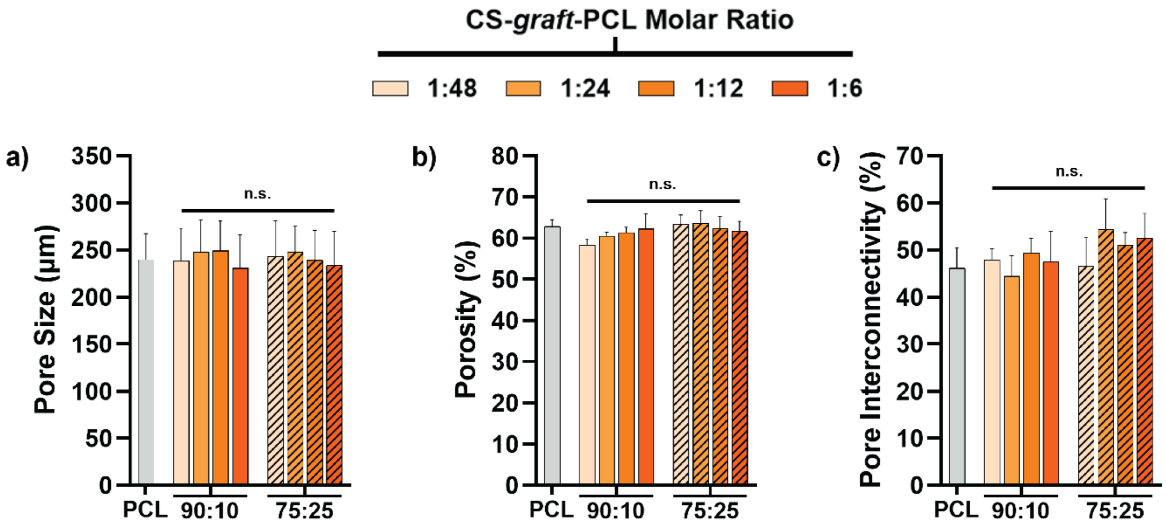

Figure S7. Scaffold (a) pore size, (b) % porosity, and (c) % pore interconnectivity. Here, "n.s." indicates no statistical significance relative to the PCL control.

Table S3. Scaffold pore size, % porosity, and % pore interconnectivity.

Composition	Pore Size (µm)	Porosity (%)	Pore Interconnectivity (%)
PCL	239.80 ± 27.37	62.88 ± 1.31	46.12 ± 4.33
**90:10 Compositions**			
1:48	238.65 ± 34.27	58.33 ± 1.25	47.95 ± 2.30
1:24	248.02 ± 33.83	60.51 ± 0.77	44.40 ± 4.38
1:12	249.18 ± 31.52	61.38 ± 1.10	49.39 ± 3.14
1:6	230.47 ± 35.56	62.29 ± 2.95	47.51 ± 6.43
**75:25 Compositions**			
1:48	243.28 ± 37.76	63.42 ± 1.80	46.57 ± 6.09
1:24	248.07 ± 27.33	63.60 ± 2.50	54.42 ± 6.42
1:12	238.96 ± 31.88	62.36 ± 2.36	51.15 ± 2.55
1:6	233.63 ± 36.04	61.63 ± 2.03	52.53 ± 5.24

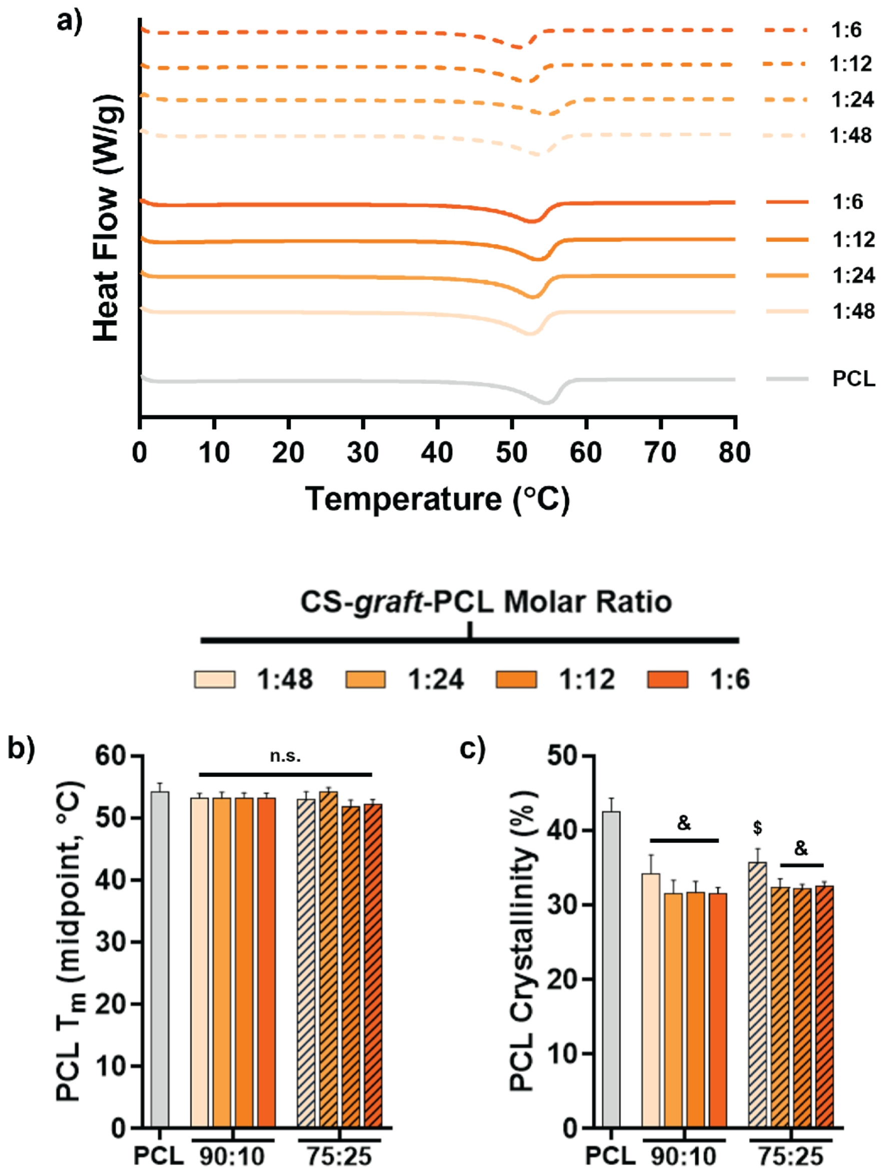

Figure S8. (a) Representative DSC thermograms of scaffolds. For PCL/PCL-*graft*-CS scaffolds, "solid" lines represent 90:10 compositions and "dashed" lines represent 75:25 compositions. (b) PCL T_m and (c) PCL % crystallinity within scaffolds. Statistical significance is indicated by symbols (where "n.s." represents no statistical significance, \$ represents $p < 0.001$, and & represents $p < 0.0001$ relative to the PCL control, respectively).

Table S4. Scaffold PCL T_m and PCL % crystallinity.

Composition	PCL T_m (onset, °C)	PCL T_m (midpoint, °C)	PCL Crystallinity (%)
PCL	48.10 ± 1.47	54.24 ± 1.32	42.56 ± 1.73
**90:10 Compositions**			
1:48	47.32 ± 0.76	53.25 ± 0.67	34.18 ± 2.56
1:24	47.56 ± 1.07	53.11 ± 1.02	31.51 ± 1.78
1:12	47.38 ± 0.74	53.24 ± 0.77	31.65 ± 1.51
1:6	47.34 ± 1.09	53.26 ± 0.71	31.53 ± 0.81
**75:25 Compositions**			
1:48	46.65 ± 1.75	53.01 ± 1.23	35.76 ± 1.81
1:24	47.18 ± 0.85	54.11 ± 0.75	32.40 ± 1.12
1:12	45.26 ± 1.08	51.86 ± 1.02	32.26 ± 0.50
1:6	45.44 ± 0.82	52.18 ± 0.81	32.54 ± 0.55

Table S5. Scaffold shape fixity (R_f) and shape recovery (R_r) for cycle 1 and cycle 2.

Composition	R_f (Cycle 1)	R_r (Cycle 1)	R_f (Cycle 2)	R_r (Cycle 2)
PCL	102.81 ± 1.26	101.99 ± 3.17	103.12 ± 6.89	98.40 ± 1.96
**90:10 Compositions**				
1:48	98.19 ± 0.19	101.93 ± 3.35	97.97 ± 1.89	101.60 ± 1.54
1:24	96.54 ± 1.25	98.81 ± 1.31	94.65 ± 0.63	100.00 ± 1.66
1:12	98.82 ± 0.97	99.11 ± 1.51	98.15 ± 1.62	100.80 ± 4.72
1:6	96.61 ± 1.83	101.90 ± 2.05	95.65 ± 0.11	99.07 ± 5.11
**75:25 Compositions**				
1:48	96.90 ± 0.80	99.59 ± 2.07	96.63 ± 0.53	101.42 ± 1.83
1:24	95.87 ± 0.79	96.58 ± 2.68	94.56 ± 0.82	100.87 ± 0.62
1:12	100.80 ± 7.57	98.25 ± 2.27	95.92 ± 0.09	99.29 ± 1.10
1:6	97.34 ± 2.30	97.68 ± 2.07	93.78 ± 2.18	100.45 ± 1.06

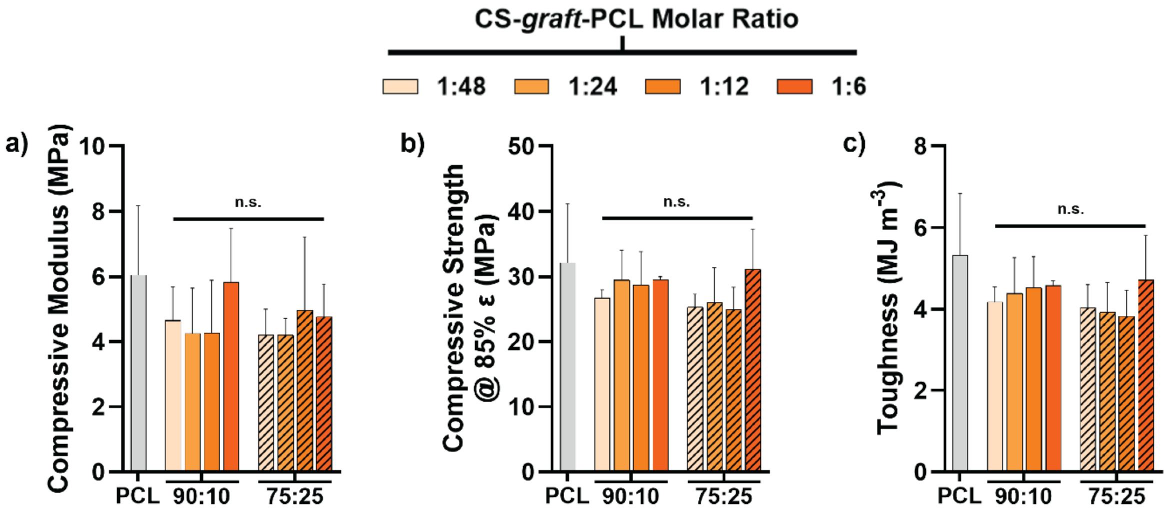

Figure S9. Compressive (a) modulus, (b) strength, and (c) toughness of scaffolds. Here, "n.s." indicates no statistical significance relative to the PCL control.

Table S6. Scaffold compressive mechanical properties.

Composition	Modulus (MPa)	Strength (MPa)	Toughness (MJ m^{-3})
PCL	6.05 ± 2.13	31.13 ± 9.07	5.32 ± 1.51
**90:10 Compositions**			
1:48	4.65 ± 1.02	26.76 ± 1.19	4.17 ± 0.37
1:24	4.25 ± 1.39	29.45 ± 4.58	4.38 ± 0.88
1:12	4.27 ± 1.61	28.72 ± 5.11	4.52 ± 0.76
1:6	5.83 ± 1.63	29.53 ± 0.44	4.58 ± 0.11
**75:25 Compositions**			
1:48	4.20 ± 0.80	25.31 ± 1.96	4.04 ± 0.55
1:24	4.20 ± 0.51	25.97 ± 5.40	3.92 ± 0.73
1:12	4.95 ± 2.27	24.90 ± 3.42	3.83 ± 0.64
1:6	4.77 ± 1.00	31.12 ± 6.17	4.71 ± 1.10

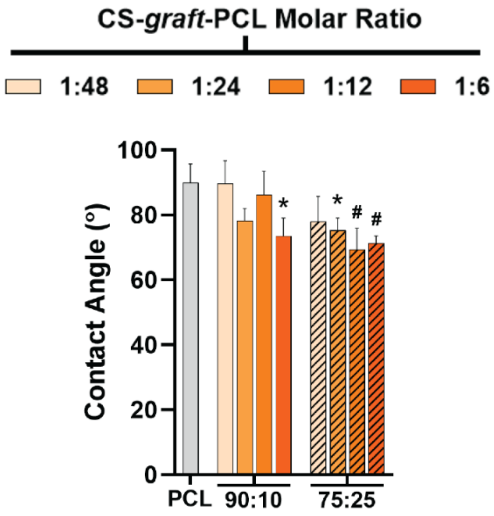

Figure S10. Static water contact angle (θ_{static}) values of films of analogous compositions to scaffolds. Statistical significance is indicated by symbols (where * represents $p < 0.05$ and # represents $p < 0.01$ relative to PCL control, respectively).

Table S7. Static water contact angle (θ_{static}) values of films of analogous compositions to scaffolds.

**Composition**	**θ_{static} (°)**
PCL	89.97 ± 4.69
**90:10 Compositions**	
1:48	89.62 ± 5.76
1:24	78.17 ± 3.07
1:12	86.17 ± 6.03
1:6	73.57 ± 4.50
**75:25 Compositions**	
1:48	77.97 ± 6.35
1:24	75.30 ± 3.08
1:12	69.33 ± 5.43
1:6	71.37 ± 1.80

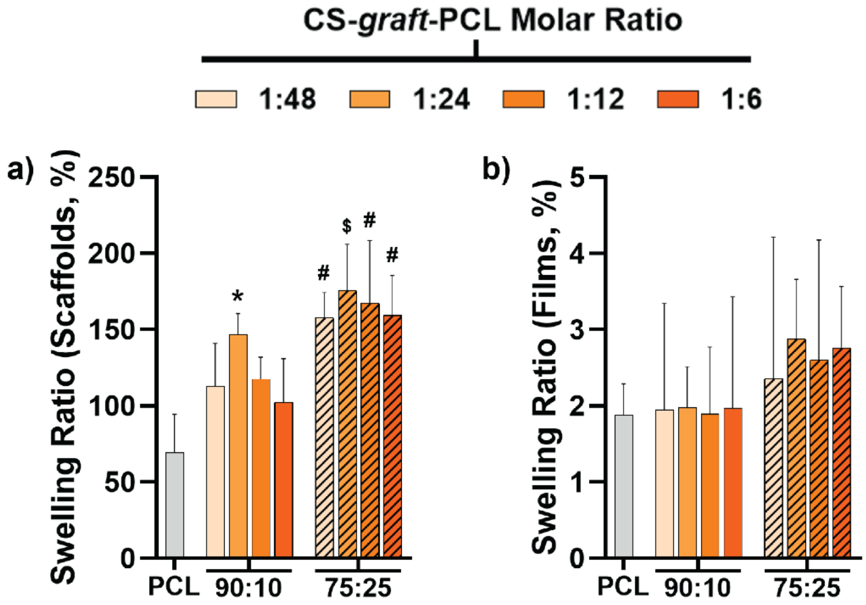

Figure S11. Swelling ratios of (a) scaffolds and (b) films. Statistical significance is indicated by symbols (where * represents $p < 0.05$, # represents $p < 0.01$, and $ represents $p < 0.001$ relative to PCL control, respectively).

Table S8. Scaffold and film swelling ratios. Samples were submerged in PBS for 7 days.

Composition	Swelling Ratio (Scaffolds, %)	Swelling Ratio (Films, %)
PCL	69.43 ± 24.95	1.88 ± 0.41
**90:10 Compositions**		
1:48	112.97 ± 27.90	1.95 ± 1.40
1:24	147.05 ± 13.41	1.98 ± 0.53
1:12	117.70 ± 14.26	1.90 ± 0.87
1:6	102.44 ± 28.42	1.97 ± 1.46
**75:25 Compositions**		
1:48	157.84 ± 16.48	2.36 ± 1.86
1:24	175.78 ± 30.21	2.88 ± 0.78
1:12	167.38 ± 41.14	2.60 ± 1.58
1:6	159.46 ± 26.10	2.76 ± 0.81

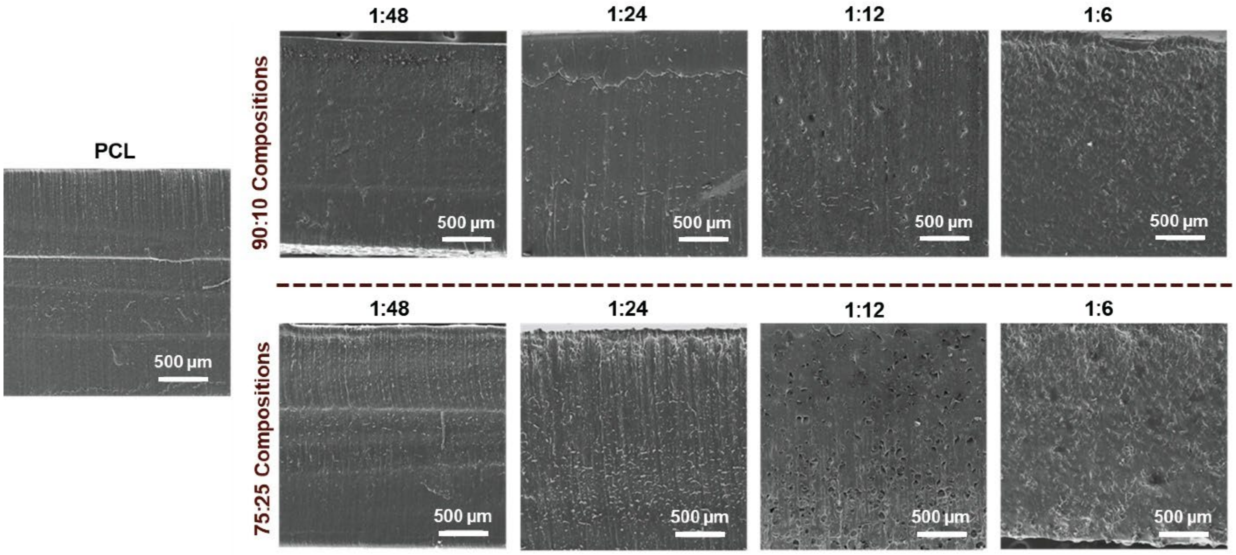

Figure S12. SEM images of film cross sections.

Table S9. Scaffold mass remaining (%) during accelerated degradation study. Scaffolds were submerged in 0.05 M NaOH at 37°C for up to 21 days.

		**Composition**							
		**90:10 Compositions**				**75:25 Compositions**			
**Days**	**PCL**	***1:48***	***1:24***	***1:12***	***1:6***	***1:48***	***1:24***	***1:12***	***1:6***
1	98.57 ± 0.14	97.91 ± 1.66	94.89 ± 2.79	97.12 ± 2.20	97.91 ± 0.89	97.91 ± 1.66	86.67 ± 3.40	96.20 ± 1.68	93.89 ± 3.59
2	98.20 ± 0.77	94.19 ± 2.12	93.67 ± 5.00	98.20 ± 0.77	95.38 ± 2.78	94.19 ± 2.12	87.00 ± 1.63	92.83 ± 2.48	92.01 ± 4.98
3	96.99 ± 2.19	97.66 ± 2.58	91.34 ± 3.10	93.58 ± 3.57	96.99 ± 2.19	96.22 ± 2.20	83.68 ± 7.42	90.74 ± 2.75	87.25 ± 12.29
4	97.46 ± 1.03	96.46 ± 0.71	85.33 ± 2.49	85.40 ± 3.10	89.91 ± 7.24	94.05 ± 1.40	79.33 ± 2.49	87.80 ± 1.65	81.78 ± 4.79
5	97.09 ± 1.12	96.75 ± 0.66	86.67 ± 0.94	90.20 ± 1.40	82.83 ± 6.24	93.38 ± 3.09	69.67 ± 12.71	86.29 ± 5.78	80.38 ± 5.54
6	96.37 ± 1.90	94.37 ± 2.93	81.67 ± 5.22	87.62 ± 6.61	81.47 ± 8.07	91.59 ± 3.28	72.14 ± 4.51	85.70 ± 3.88	79.33 ± 7.36
7	95.89 ± 2.16	97.97 ± 2.87	81.00 ± 2.16	83.43 ± 7.91	76.00 ± 4.90	90.35 ± 3.38	67.33 ± 9.46	76.70 ± 4.96	79.00 ± 2.20
10	87.09 ± 1.87	87.54 ± 1.97	79.27 ± 5.47	76.67 ± 2.62	74.00 ± 4.32	83.44 ± 3.86	55.43 ± 8.77	72.63 ± 5.19	64.67 ± 7.16
12	81.97 ± 6.80	79.84 ± 7.26	70.00 ± 1.63	78.00 ± 2.16	65.67 ± 4.19	71.24 ± 6.69	35.97 ± 4.80	47.27 ± 4.18	42.33 ± 3.22
15	81.53 ± 6.34	78.43 ± 10.62	54.00 ± 6.98	74.17 ± 1.55	63.03 ± 3.70	59.17 ± 6.58	32.03 ± 4.95	38.53 ± 2.90	44.07 ± 8.73
18	68.07 ± 1.92	70.87 ± 3.27	48.33 ± 7.41	62.83 ± 2.25	51.67 ± 7.41	59.54 ± 11.89	12.50 ± 8.00	28.10 ± 4.51	24.13 ± 8.92
21	63.07 ± 5.54	65.34 ± 5.89	28.90 ± 14.24	40.10 ± 5.78	41.75 ± 9.25	38.80 ± 2.71	0.00 ± 0.00	24.03 ± 6.82	10.10 ± 5.55

Table S10. Direct contact assays — Normalized ATP bioluminescence values of *C. albicans* biofilm formation on PCL and PCL/CS-*graft*-PCL films (24 h).

Composition	Direct Contact
PCL	100.00 ± 10.85
**90:10 Compositions**	
1:48	56.98 ± 19.52
1:24	25.35 ± 6.28
1:12	24.16 ± 2.48
1:6	29.36 ± 5.26
**75:25 Compositions**	
1:48	24.42 ± 2.60
1:24	34.31 ± 2.72
1:12	14.44 ± 5.63
1:6	22.32 ± 5.83

Table S11. Indirect contact assays — Normalized crystal violet absorbance values of *C. albicans* biofilm growth following incubation of growth media (24 h) with films, and the resulting leachate/extract-containing growth media sequentially collected, inoculated, and an aliquot placed in the well of a polystyrene plate (24 h).

Composition	Indirect Contact
PCL	100 ± 3.55
**90:10 Compositions**	
1:48	58.77 ± 6.08
1:24	17.54 ± 3.93
1:12	7.67 ± 1.30
1:6	6.91 ± 2.91
**75:25 Compositions**	
1:48	41.86 ± 9.86
1:24	12.01 ± 2.26
1:12	7.79 ± 1.35
1:6	8.01 ± 3.35

References

(1) Fernandez-Megia, E.; Novoa-Carballal, R.; Quiñoá, E.; Riguera, R. Optimal routine conditions for the determination of the degree of acetylation of chitosan by 1H-NMR. *Carbohydr. Polym.* **2005**, *61* (2), 155-161.

(2) Hirai, A.; Odani, H.; Nakajima, A. Determination of degree of deacetylation of chitosan by 1H NMR spectroscopy. *Polym. Bull.* **1991**, *26* (1), 87-94.

(3) Yu, H.; Wang, W.; Chen, X.; Deng, C.; Jing, X. Synthesis and characterization of the biodegradable polycaprolactone-graft-chitosan amphiphilic copolymers. *Biopolymers* **2006**, *83* (3), 233-242.

(4) Duan, K.; Chen, H.; Huang, J.; Yu, J.; Liu, S.; Wang, D.; Li, Y. One-step synthesis of amino-reserved chitosan-graft-polycaprolactone as a promising substance of biomaterial. *Carbohydr. Polym.* **2010**, *80* (2), 498-503.

(5) Shirzaei Sani, I.; Rezaei, M.; Baradar Khoshfetrat, A.; Razzaghi, D. Preparation and characterization of polycaprolactone/chitosan-g-polycaprolactone/hydroxyapatite electrospun nanocomposite scaffolds for bone tissue engineering. *Int. J. Biol. Macromol.* **2021**, *182*, 1638-1649.
